# Menthol can be safely applied to improve thermal perception during physical exercise: a meta-analysis of randomized controlled trials

**DOI:** 10.1038/s41598-020-70499-9

**Published:** 2020-08-12

**Authors:** Patrik Keringer, Nelli Farkas, Noemi Gede, Peter Hegyi, Zoltan Rumbus, Zsolt Lohinai, Margit Solymar, Kasidid Ruksakiet, Gabor Varga, Andras Garami

**Affiliations:** 1 Department of Thermophysiology, Institute for Translational Medicine, Medical School, University of Pecs, 7624 Pecs, Hungary; 2 Institute for Translational Medicine, Szentagothai Research Centre, Medical School, University of Pecs, 7624 Pecs, Hungary; 3 Institute of Bioanalysis, Medical School, University of Pecs, 7624 Pecs, Hungary; 4 Department of Translational Medicine, First Department of Medicine, Medical School, University of Pecs, 7624 Pecs, Hungary; 5 Department of Conservative Dentistry, Faculty of Dentistry, Semmelweis University, 1088 Budapest, Hungary; 6 Department of Oral Biology, Faculty of Dentistry, Semmelweis University, 1089 Budapest, Hungary

**Keywords:** Medical research, Homeostasis, Energy metabolism, Fatigue, Fever

## Abstract

Menthol is often used as a cold-mimicking substance to allegedly enhance performance during physical activity, however menthol-induced activation of cold-defence responses during exercise can intensify heat accumulation in the body. This meta-analysis aimed at studying the effects of menthol on thermal perception and thermophysiological homeostasis during exercise. PubMed, EMBASE, Cochrane Library, and Google Scholar databases were searched until May 2020. Menthol caused cooler thermal sensation by weighted mean difference (WMD) of − 1.65 (95% CI, − 2.96 to − 0.33) and tended to improve thermal comfort (WMD = 1.42; 95% CI, − 0.13 to 2.96) during physical exercise. However, there was no meaningful difference in sweat production (WMD = − 24.10 ml; 95% CI, − 139.59 to 91.39 ml), deep body temperature (WMD = 0.02 °C; 95% CI, − 0.11 to 0.15 °C), and heart rate (WMD = 2.67 bpm; 95% CI − 0.74 to 6.09 bpm) between the treatment groups. Menthol improved the performance time in certain subgroups, which are discussed. Our findings suggest that different factors, viz., external application, warmer environment, and higher body mass index can improve menthol’s effects on endurance performance, however menthol does not compromise warmth-defence responses during exercise, thus it can be safely applied by athletes from the thermoregulation point of view.

## Introduction

Menthol (2-isopropyl-5-methylcyclohexanol) is a lipophilic, organic compound which can be extracted from essential oils of aromatic plants or produced synthetically^1,2^. The most common naturally occurring form of menthol is the l-isomer, which is used in various products, e.g., candies, beverages, cigarettes, and toothpastes, mainly because of its cooling, analgesic, and anti-inflammatory effects^2,3^. It has long been assumed that menthol might improve different aspects of physical performance such as endurance, speed, strength, and joint range of motion, consequently it is often used by athletes in the form of sprays, creams, tapes, beverages, etc.^4,5^.

Warming-up before an exercise is often used to optimize muscle temperature and, thereby, maximal muscle power production, however, at high ambient temperatures (T_a_), it increases the thermal and circulatory strain^6^. Endurance exercise capacity at a high T_a_ is impaired by heat stress prior to exercise^7^, and hyperthermia induces fatigue during short intense activities and prolonged exercise in the heat^8^. On the contrary, physical cooling of the body before and during exercise in the warmth improves exercise endurance and reduces cardiovascular strain^9^. A recent meta-analysis of 45 studies also concluded that physical cooling improves aerobic and anaerobic exercise performance in hot conditions^10^. From animal experiments it is known that the transient receptor potential (TRP) melastatin-8 (M8) channel, formerly called as menthol receptor, is a universal cold sensor in the thermoregulation system. The pharmacological modulation of TRPM8 with systemic (intravenous or intraperitoneal) administration of an antagonist changes the activity of the cold-activated neural pathway^11^, which raises the possibility that activation of TRPM8 with ligand agonists like menthol can have similar effects to physical cooling before or during physical exercise. Indeed, in exercising humans menthol administration resulted in increased thermogenesis^12^, decreased sweating^13,14^, and more pronounced skin vasoconstriction^15,16^, consequently in elevated deep body temperature (T_b_)^17,18^; that is the same pattern of thermoregulatory effector recruitment which can be observed as part of the cold-defence responses^19^. Importantly, the menthol-induced decrease in heat loss and elevation in deep T_b_ can increase the risk for heat exhaustion and adverse cardiovascular events in the warmth^13,17^, therefore, the safety of menthol application in physical exercise, especially at high T_a_, remains questionable. In contrast with the aforementioned studies showing an increased risk for the onset of heat-related illnesses in association with menthol application, several human studies showed beneficial effects of menthol on physiological, psychological, and performance parameters during physical exercise^18,20–27^, while a decent number of studies found no effect^28–31^.

The observed discrepancies among the studies may originate from differences in study designs, application methods (route of administration, dosage, location of the administration, and the surface area), and experimental conditions (e.g., T_a_). Menthol-containing products can be administered externally (e.g., in spray or gel form) or internally (e.g., mouth rinse, beverage consumption). External application has been shown to be more beneficial than the internal in sports physiology and on endurance performance^13,14,23,25^, whereas other authors found that internally applied menthol is more effective^20–22,24,27^, and yet others showed no effect of menthol independently from the application method^26,28–31^.

In our meta-analysis, we analysed how menthol administration affects the changes in perceptual and physiological parameters of thermoregulation, and in indicators (viz., power output and performance time) of the overall endurance performance during physical exercise in healthy humans.

## Methods

Our meta-analysis was conducted in accordance with the guidelines of the Preferred Reporting Items for Systematic Reviews and Meta-Analysis (PRISMA) protocols^32^ (Supplementary Table S1). The analysis was based on the Participants, Intervention, Comparison, and Outcome model: in physically active, healthy participants, we investigated the effects of menthol application compared to controls (i.e., no menthol or placebo treatment) on physiological and perceptual parameters and on indicators of endurance performance during physical exercise. The protocol for this meta-analysis was registered on PROSPERO (registration number: CRD42019125034).

### Search strategy

A search of the PubMed, EMBASE, and Cochrane Controlled Trials Registry databases was performed until May 2020 using the following search key: “(menthol OR mint OR peppermint OR mentha OR spearmint) AND (temperature OR “heart rate” OR “oxygen uptake” OR lactate OR “sweat rate” OR “physical performance” OR exhaustion)”. We restricted our search to randomized controlled human trials published in English without time period limitations. A manual search of the reference lists of identified full-text articles was also performed in Google Scholar for eligible studies. The search was conducted separately by two authors (PK, AG), who also assessed study eligibility and extracted data from the selected studies independently. Disagreements were resolved, if needed, by a third party (ZR).

### Study selection and data extraction

After screening on the titles and abstracts of the identified publications, the full texts of eligible articles were obtained. We included studies which reported at least one of the following values: thermal sensation (TS), thermal comfort (TC), T_b_, sweat production, heart rate, performance time, and power output in menthol-treated and control healthy subjects before and during physical exercise. For all parameters, the maximal change from baseline after menthol treatment (and the corresponding value at the same time point in the control group) was extracted to assess the acute effect of menthol. In each study we calculated the difference between the menthol-treated and control groups, which was then included in the analyses. This approach allowed for taking into considerations differences in experimental protocols between studies. From all included articles, we extracted the group size, the reported mean values and standard deviations (SD) of the parameters of interest, and the level of statistical significance (p value). To analyse the effects of menthol under different conditions, we also divided the studies into subgroups, which were determined on the basis of known influencing factors^33,34^, and data availability. The main influencing factors were grouped in three categories: characteristics of the subject (body mass index [BMI] and heat acclimation), study protocol (trial type and menthol administration method), and environmental circumstances (airflow and T_a_).

### Statistical analysis

In each study, we calculated the maximum change in the outcome parameter from baseline after menthol application and the change from baseline until the same time point in the control group. Then, we calculated the weighted mean difference (WMD) with 95% confidence interval (CI) in the change of the parameter between the menthol-treated and the control groups. The statistical analysis was performed according to the standard methods of meta-analysis by using a random effects model. The effects were considered significant when p < 0.05. Using both p value and CI allowed us to detect physiologically relevant differences between the groups even in the case of overlapping CIs^35^.

To study perceptual responses, data on TS and TC were collected. By definition, TS identifies the relative intensity of the temperature being sensed, and, as such, provides the body with information about the thermal environment, while TC means subjective indifference with the thermal environment, so that thermal pleasure is perceived when a stimulus aims to restore TC (for a comprehensive review, see Flouris & Schlader^36^). In the analysed studies, TS scales were used with ranges of 7-point^25^, 9-point^22^, and 20-point^14,29,30^, while in case of TC, the authors used 4-point^25^, 7-point^18,22,23^, and 20-point ranges^14,29,30^. Since TS and TC were determined by different visual analogue scales in the studies, in order to make the reported TS and TC values comparable for our meta-analysis, while also minimizing the need for conversion of the originally reported data in the studies, if required, the reported values were extrapolated into a unified scale, ranging from 0 to 20. The scales were bilateral, i.e., 0 corresponded to neutral, in several studies. For example, TS was assessed with a 9-point scale ranging from very cold (− 4) to neutral (0) to very hot (4). Thus, after extrapolation of the endpoints of the original scales to 0 and 20, in case of TS the middle of the unified scale (10) represented the neutral situation and an increase in TS between 0 (very cold) and 20 (very hot) indicated a weaker cold or stronger warmth sensation. To assess TC, three types of scales were used in the analysed studies: 20-cm visual analogue scale (0: very uncomfortable, 20: very comfortable)^14,29,30^, 7-point scale (− 3: much too cool, 0: comfortable, 3: much too warm)^18,22,23^, and 4-point scale (1: comfortable, 4: very uncomfortable)^25^. In the studies using 7-point scale, we did not find any value smaller than 0, thus we considered the scale from 0 (comfortable) to 3 (uncomfortable) and these endpoints were extrapolated to 20 and 0, respectively. In the 4-point scale, the 1 (comfortable) and 4 (uncomfortable) endpoints were extrapolated to 20 and 0, respectively. As result, the unified 20-point scale, ranging from very uncomfortable to very comfortable, resembled the one used in the pioneer study by Gagge et al.^37^. In this scale, a higher TC value between 0 (very uncomfortable) and 20 (very comfortable) corresponded to more pleasant comfort feeling.

With regards to performance time, it must be noted that depending on the exercise protocol its decrease and increase can both indicate an improved endurance performance. In time-to-exhaustion (TTE) protocols, a longer performance time indicates improved endurance as the subjects are able to perform the exercise for a longer time period, whereas in time-trial (TT) protocols the subjects aim at finishing a predefined exercise task as fast as they can, thus longer performance time indicates reduced endurance in these tasks. Therefore, in our analyses, we always separated the TTE and TT protocols in different groups when the investigated outcome was performance time, similarly as in previous studies^33,38^.

Inter-study heterogeneity was tested with the Q homogeneity test and with the I^2^ statistical test, where I^2^ is the proportion of total variation attributable to between-study variability (an I^2^ value of more than 50% was considered as an indication of considerable heterogeneity). Publication bias was assessed by Egger’s test and visual inspection of funnel plots (Supplementary Figs. S7-S10). To evaluate the quality of the included trials, two independent reviewers (PK and ZR) assessed the risk of bias according to the Cochrane Handbook^39^. The methodology described for random sequence generation, allocation concealment, blinding of participants and personnel, blinding of outcome assessment, completeness of outcome data, and selective outcome reporting was assessed (Supplementary Table S2), similarly as in our recent study^40^.

All analyses were performed using the Comprehensive Meta-Analysis software (version 3.3; Biostat, Inc., Engelwood, NJ).

## Results

### Study selection and characteristics

**Figure 1:**
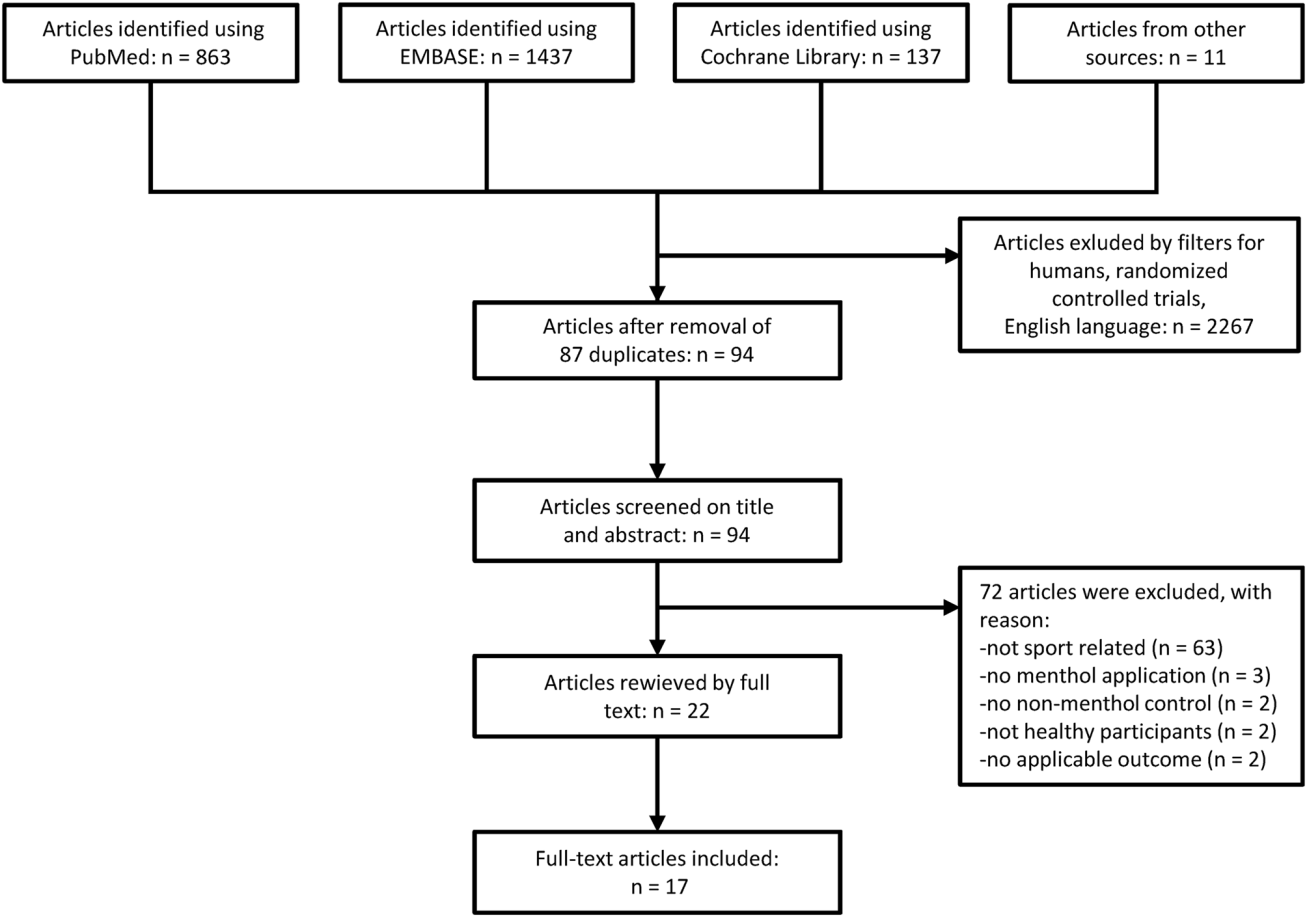
Flowchart of study selection and inclusion.

The flowchart of study selection is presented in Fig. 1. Until May 2020, a total of 2,448 records were retrieved from the PubMed (n = 863), EMBASE (n = 1,437), and Cochrane (n = 137) databases and 11 records from other sources (e.g., Google Scholar). After removing duplicates and enabling filters for human studies, randomized controlled trials, and English language, 94 articles remained. By screening on title and abstract, further 72 records were excluded from the analysis because (1) the required outcome parameters were not reported, (2) menthol-treated or control group was absent, (3) not only healthy participants were recruited, and (4) no original data was reported. The full texts of 22 articles were reviewed in detail, from which 17 papers provided eligible data for qualitative and quantitative analyses^13,14,17,18,20–31,41^. All included studies had randomized, crossover design and included data from a total of 177 athletes. The participant characteristics in the studies are presented in Supplementary Table S3. The majority of the studies was conducted in males, except for an article which included participants of both sexes^31^ and possibly another one which did not report the sex ratio in the sample^26^. In 12 trials, the participants were refrained from strenuous exercise, alcohol and caffeine intake before the experiments^13,14,20–23,25,27–30,41^, two studies included unspecified training limitations^18,24^, and three articles did not report any limitations^17,26,31^.

The mean exercise duration was 30 min in TT tests, ranging from 1 min^26^ to 71 min^28^, while in TTE protocols, it ranged between 1 min^41^ and 61 min^20^, with an average of 27 min. In two studies the subjects exercised for a fixed time duration of 45 min^17^ or 2 × 20 min^18^, while the exercise duration was calculated from distance and speed in one of the studies^26^. In all studies we considered the beginning of the exercise as the baseline, which was before the menthol treatment, except for two studies, in which menthol administration was before the start of the trial^23,25^. It should be noted that the TTE test was preceded by 45-min exercise without interruption in the study by Barwood et al.^14^, during which the subjects were repeatedly treated with menthol, thus we considered the beginning of the 45-min preliminary fatiguing task as the baseline for TS, TC, and deep T_b_. The TTE test was also preceded by physical exertion and repeated menthol administration in the study by Saldaris et al.^41^, but in that study the 3 × 30 min trials were interrupted by breaks and resting between the trials, as well as, before the TTE test. Therefore, we considered the beginning of the TTE test as the baseline in that study.

The used doses of menthol varied due to differences in administration method (i.e., internal or external), concentration (0.01–8%), volume (25–500 ml), and surface of the treated body area (4–91%). The most commonly used concentrations and volumes were, respectively, 0.01% and 25 ml in internal, while 0.2% and 100 ml in external administration routes. The details of the used administration routes, doses, and experimental procedures are summarised in Supplementary Table S4.

### Perceptual responses

**Figure 2:**
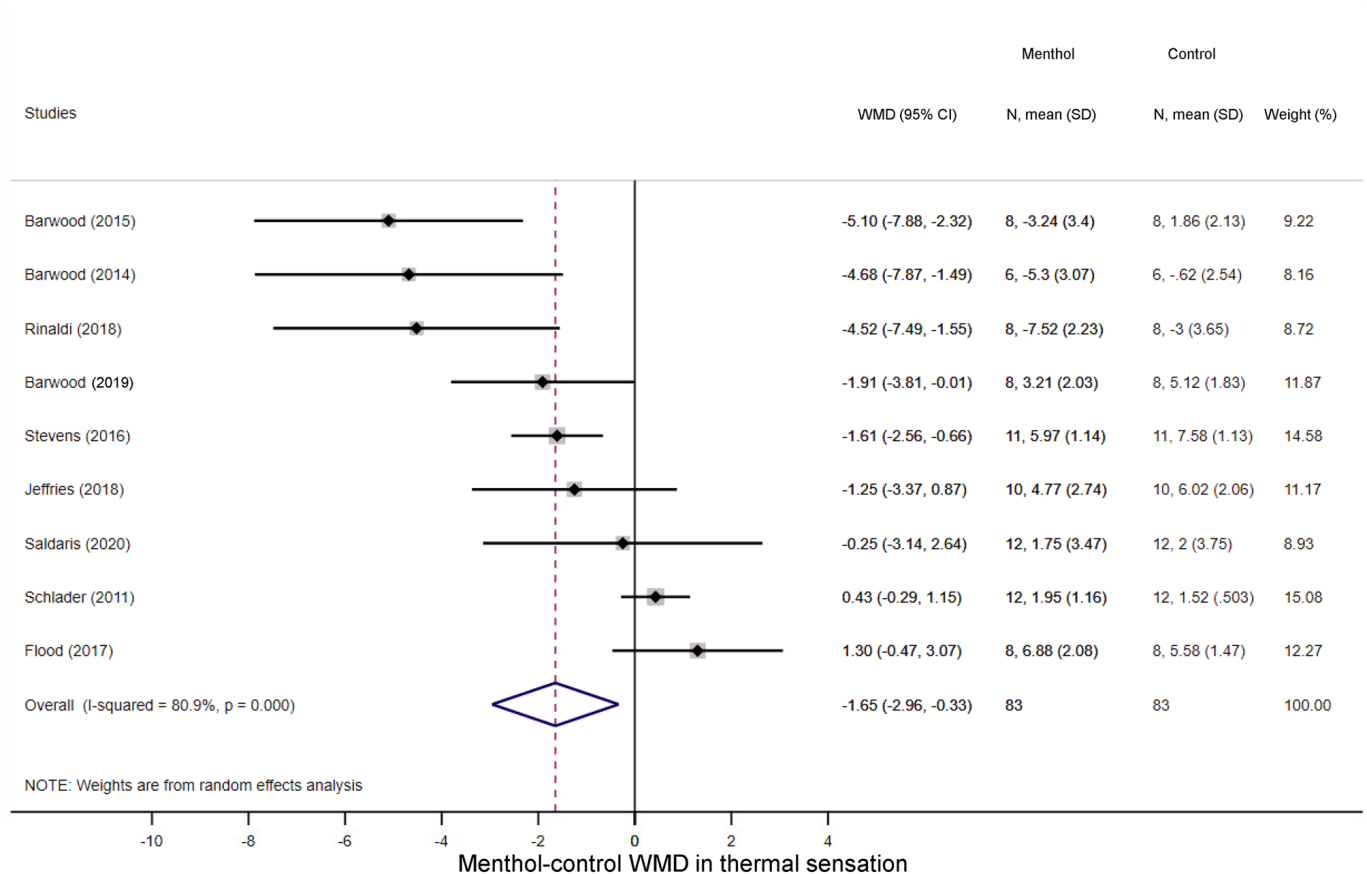
Forest plot of the weighted mean differences (WMDs) showing the effect of menthol on thermal sensation during exercise. Here, and in Figs. 3, 4, 5, 6, 7, and 8, black circles represent the WMD for each study, while the left and right horizontal arms of the circles indicate the corresponding 95% confidence intervals (CI) for the WMD. The size of the grey box is proportional to the sample size; bigger box represents larger sample size, thus bigger relative weight of the study. The diamond represents the average WMD calculated from the WMDs of the individual studies. The left and right vertices of the diamond represent the 95% CI of the average WMD. The vertical dashed line is determined by the low and top vertices of the bottom diamond and indicates the value of the average WMD of all studies in the forest plot. A WMD lesser than 0 indicates that the thermal sensation value (intensity of cold sensation) is higher in menthol-treated group, whereas a WMD higher than 0 indicates that thermal sensation is higher in control group.

**Figure 3:**
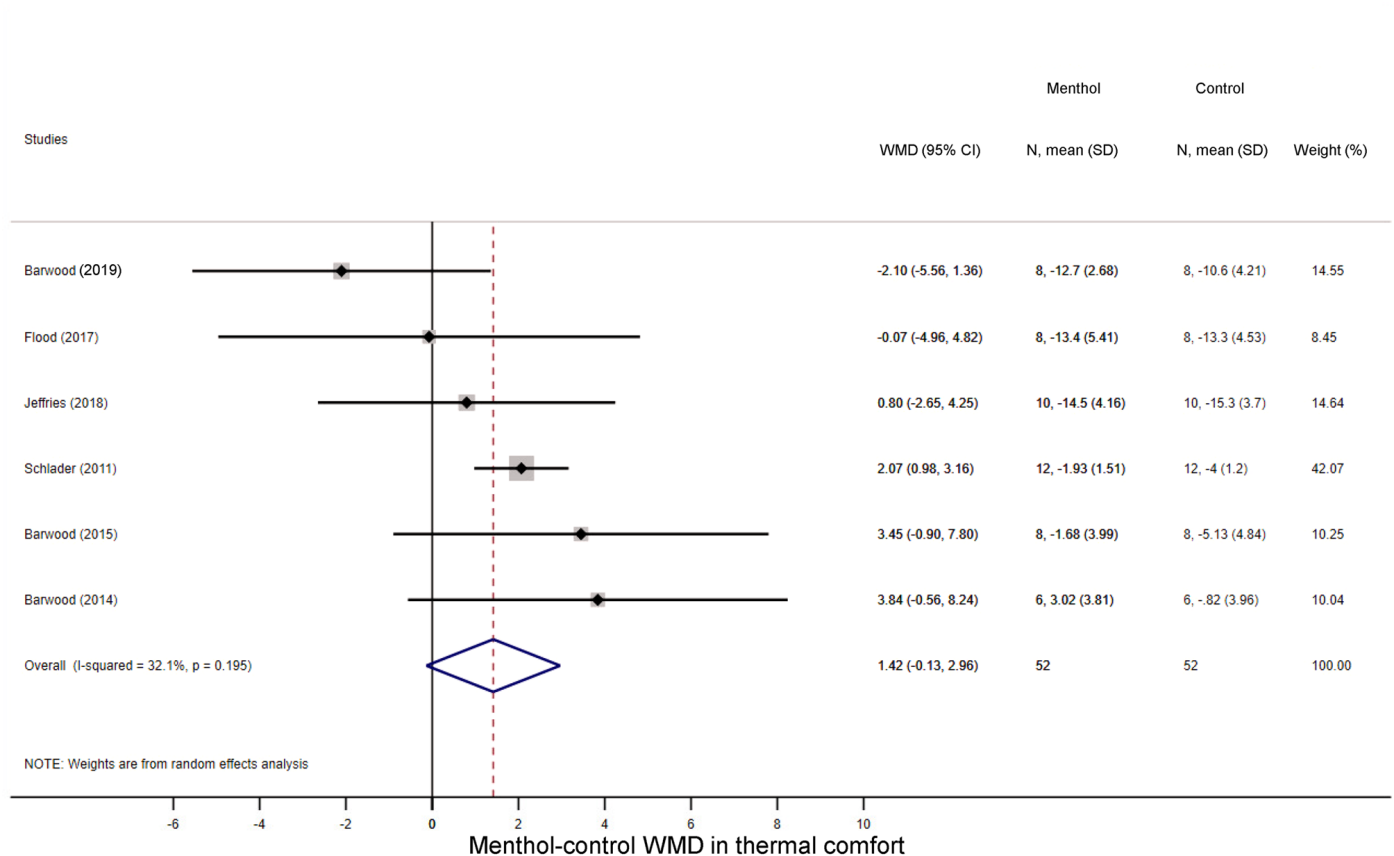
Forest plot of the weighted mean of differences (WMDs) for thermal comfort showing the effect of menthol during exercise.

**Figure 4:**
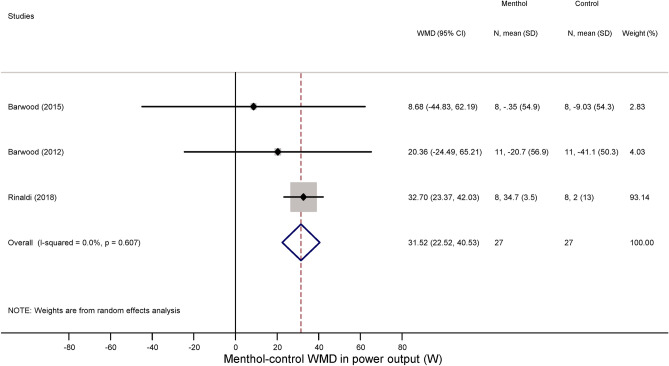
Forest plot of the weighted mean of differences (WMDs) for power output showing the effect of menthol during exercise.

First, we studied how menthol application influences perceptual responses, viz., TS and TC during exercise. As it could be expected based on to the cold-mimicking effect of menthol-containing products^1^, the TS score decreased in the menthol-treated groups as compared to controls in seven studies^14,21,22,25,29,30,41^, while two studies reported a slight increase in TS^23,25^. Accordingly, the overall WMD between the menthol-treated and control groups was − 1.65 (95% CI, − 2.96 to − 0.33; p = 0.014) (Fig. 2). The TC score decreased during physical exercise compared to baseline in all groups, but the magnitude of the decrease tended to be smaller in the menthol-treated group than in controls by a WMD of 1.42 (95% CI, − 0.13 to 2.96; p = 0.073) (Fig. 3), which indicates that the perceived temperature was more comfortable (i.e., not so hot) after menthol administration compared to controls. In subgroup analysis, we found that the absence of airflow further decreased the TS in the menthol-treated group (WMD = − 2.86; 95% CI, − 4.51 to − 1.22), whereas the menthol-induced drop was not significant when the fan was used (WMD = − 1.12; 95% CI, − 3.10 to 0.86) (Supplementary Fig. S1). The TS-decreasing effect of menthol differed significantly (p < 0.001) between the two subgroups. We also analysed whether the menthol-induced improvements in TS and TC are associated with an increased power output (an indicator of exercise intensity) during physical exercise and found that the power output remained higher in the menthol-treated groups compared to controls in all of the individual studies^18,28,30^, and accordingly, their overall average was also higher by a WMD of 31.52 W (95% CI, 22.52 to 40.53 W) (Fig. 4).

### Thermophysiological responses

**Figure 5:**
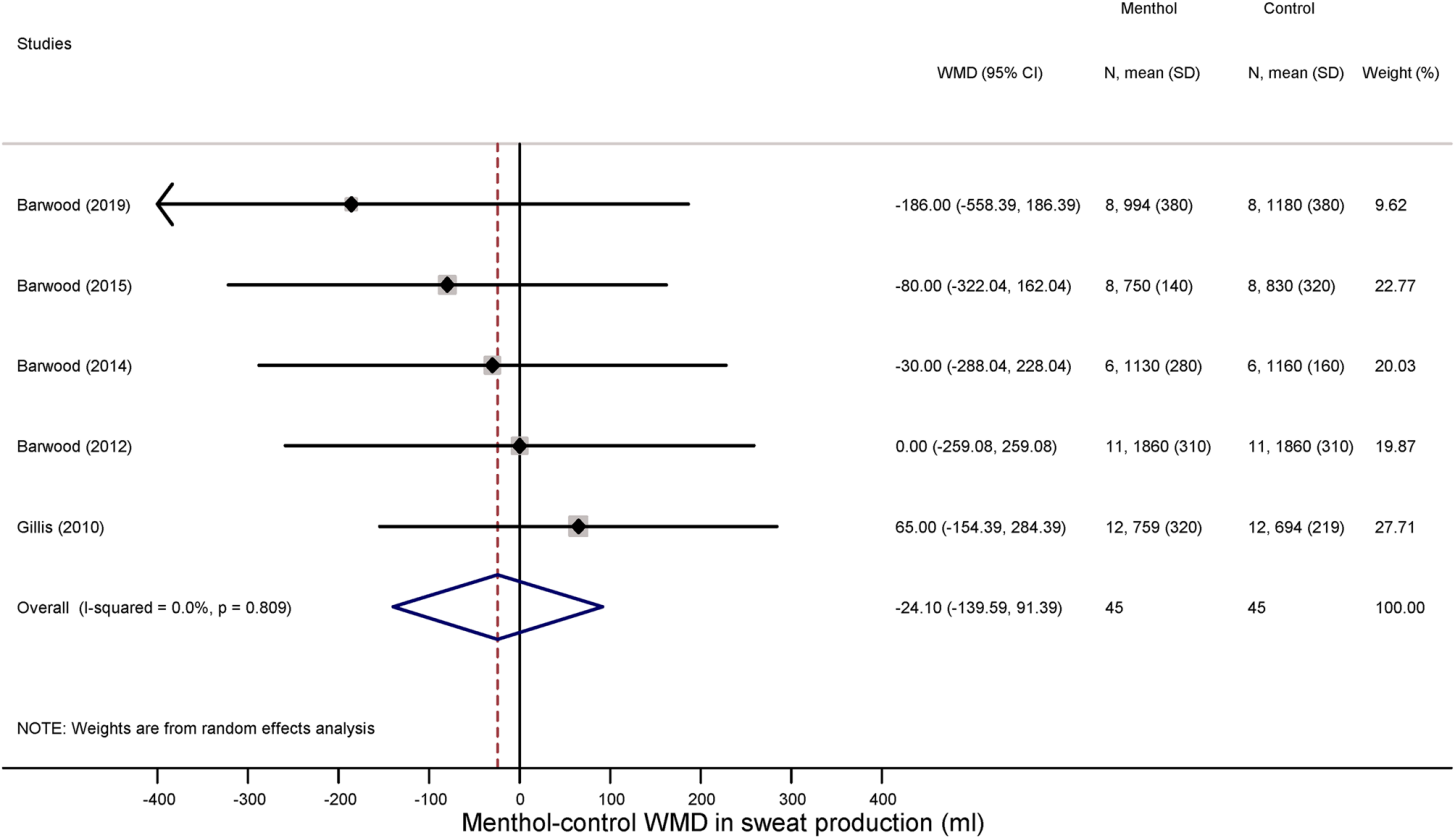
Forest plot of the weighted mean of differences (WMDs) for sweat production showing the effect of menthol during exercise.

**Figure 6:**
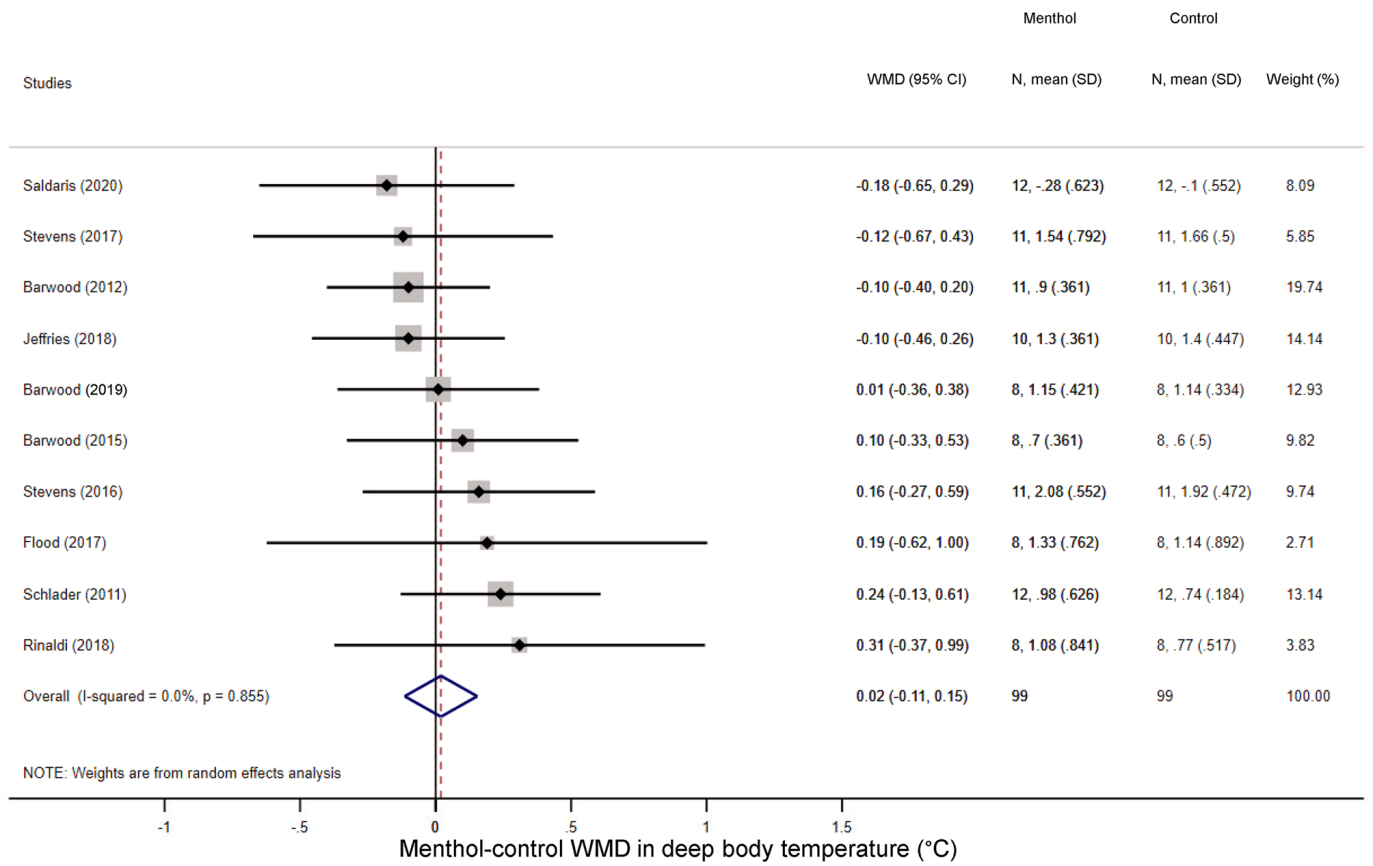
Forest plot of the weighted mean of differences (WMDs) for deep body temperature showing the effect of menthol during exercise.

**Figure 7:**
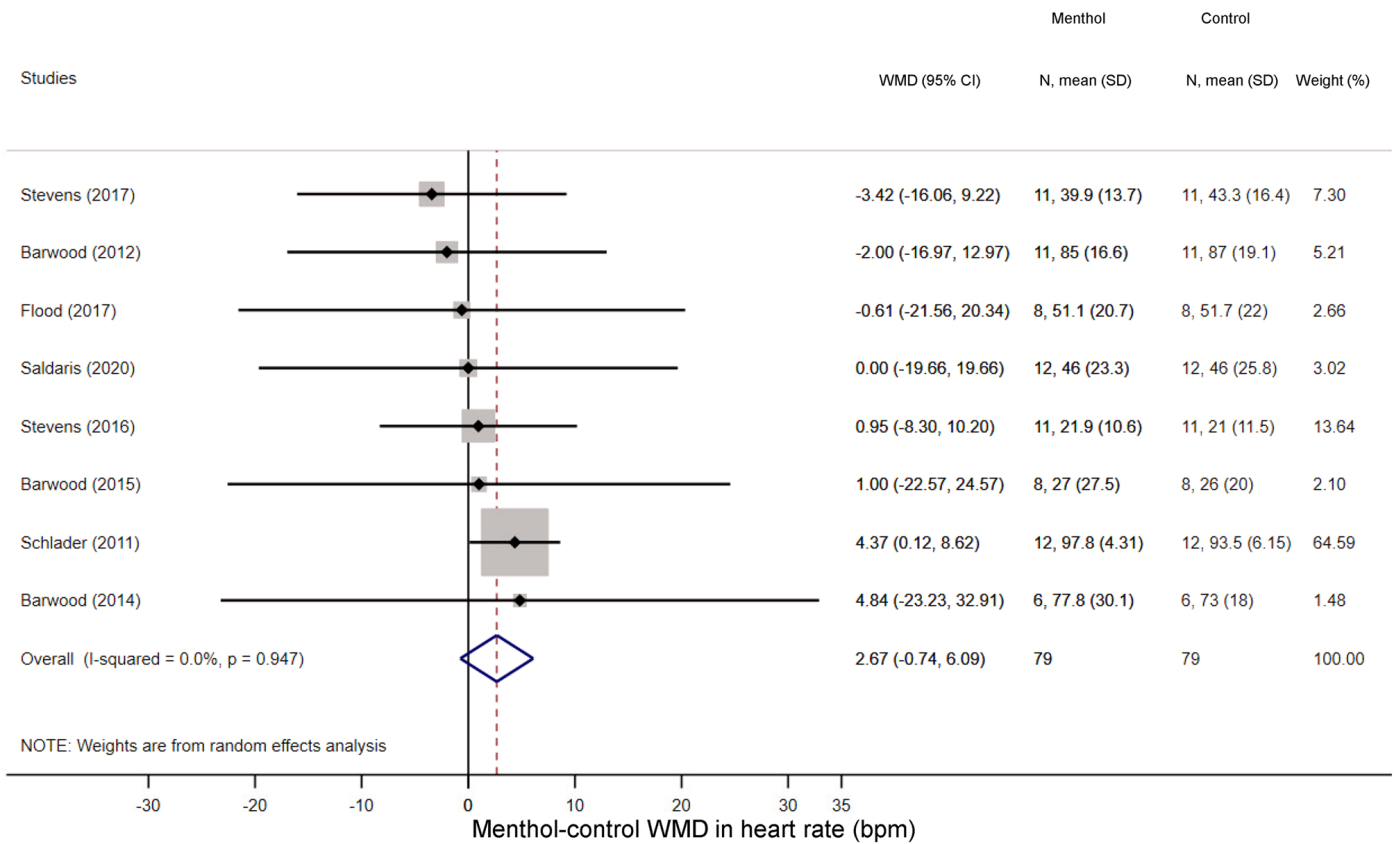
Forest plot of the weighted mean of differences (WMDs) for heart rate showing the effect of menthol during exercise.

We could extract sufficient data for the analysis of three thermoregulatory parameters: sweat production (an indicator of the activity of autonomic heat-dissipating mechanisms), heart rate (a nonspecific indicator of metabolic rate), and deep T_b_ (i.e., the tightly controlled parameter in thermoregulation). We found that the volume of sweat production did not differ significantly between the menthol-treated and control groups during exercise (WMD = − 24.10 ml; 95% CI, − 139.59 to 91.39 ml) (Fig. 5). Similar to sweat production menthol also did not have a meaningful effect on the exercise-induced increase in deep T_b_ compared to the control group (WMD = 0.02 °C; 95% CI, − 0.11 to 0.15 °C) (Fig. 6). Furthermore, there was no significant difference in exercise-induced elevation of heart rate between the treatment groups (WMD = 2.67 bpm; 95% CI − 0.74 to 6.09 bpm) (Fig. 7).

### Performance time

**Figure 8:**
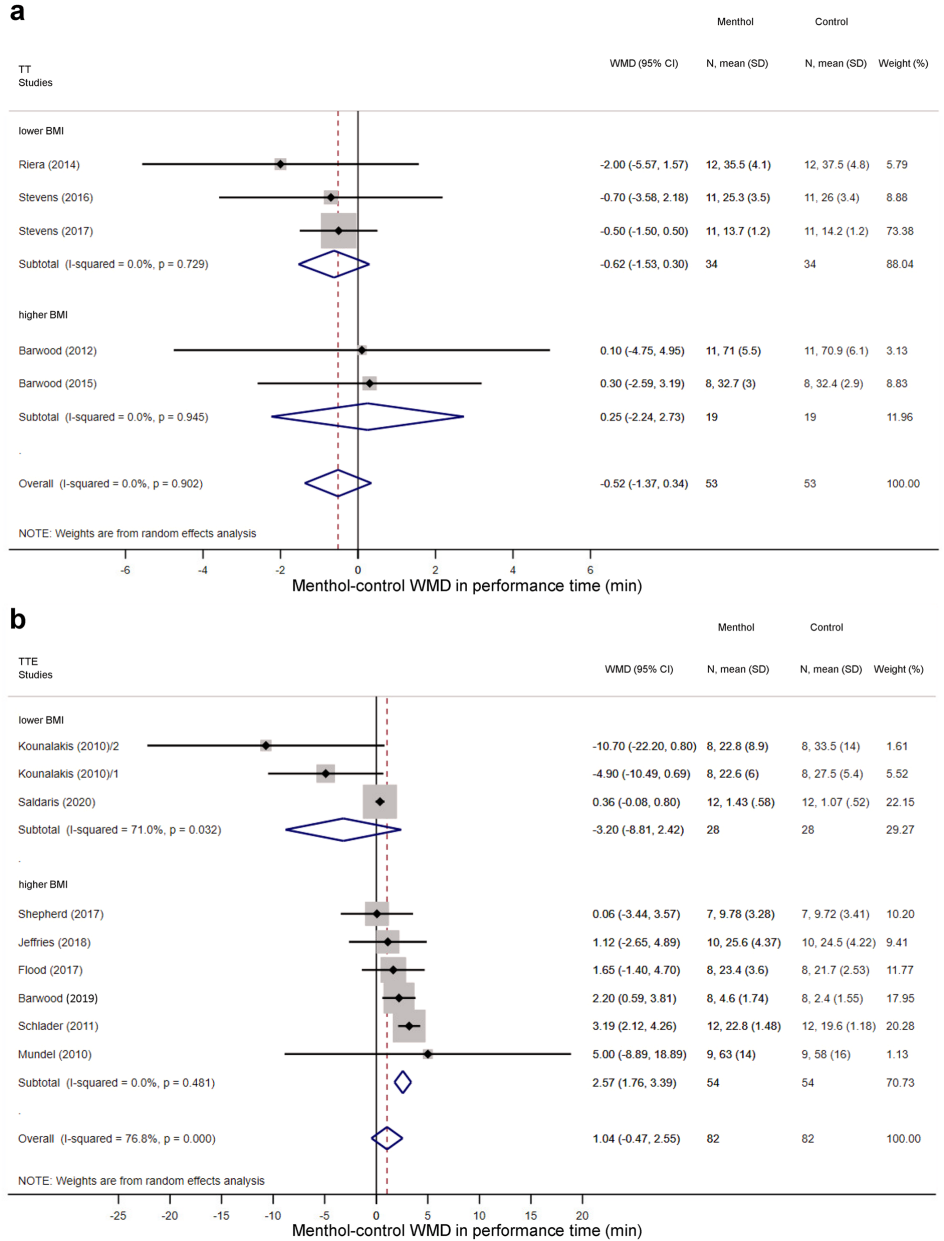
Forest plot of the weighted mean of differences (WMDs) for performance time showing the effect of menthol in (**a**) time-trial (TT) and (**b**) time-to-exhaustion (TTE) tests of athletes with lower (< 23.5) and higher (> 23.5) body mass index (BMI). The diamonds in the panels represent the average WMD calculated from the WMDs of the individual studies in each subgroup (top and middle) or in all studies (bottom).

Overall, the performance time did not differ statistically between menthol-treated and control groups in TT protocols (WMD = − 0.52 min; 95% CI, − 1.37 to 0.34 min) (Supplementary Fig. S2a) and TTE tests (WMD = 1.04 min; 95% CI, − 0.47 to 2.55 min) (Supplementary Fig. S2b). In the TT protocols, no meaningful difference was observed in the effect of menthol between subgroups of higher (above 23.5) BMI and lower (21.4–23.5) BMI (Fig. 8a). However, in the TTE tests, among athletes with higher BMI, performance time increased significantly in the menthol-treated group compared to controls (WMD = 2.57 min; 95% CI 1.76 to 3.39 min), whereas menthol tended to decrease performance time in the lower BMI group (WMD = − 3.20 min; 95% CI − 8.81 to 2.42 min) (Fig. 8b). The WMD between the treatment groups was markedly bigger in the higher than in the lower BMI subgroup (p < 0.001). We also analysed whether acclimation of the subjects to exercising in warmth influences the effects of menthol. In TT protocols, we did not find meaningful difference in menthol’s effect on performance time between non-acclimated and acclimated participants (WMD = 0.25 min; 95% CI − 2.24 to 2.73 min versus WMD = − 0.62 min; 95% CI, − 1.53 to 0.30 min) (Supplementary Fig. S3a). In TTE protocols, all of the studies were performed in non-acclimated participants and the effect of menthol was also not significant in the group (WMD = 1.13 min; 95% CI, − 0.52 to 2.77 min) (Supplementary Fig. S3b). When we compared the effect of external and internal menthol application on endurance performance, we found that external application of menthol markedly increased performance time compared to internal application in TTE exercise protocols (WMD = 0.83 min; 95% CI − 1.95 to 3.60 min versus 0.40 min, 95% CI, − 0.03 to 0.83 min; p < 0.001), while in the other subgroups no significant effect was detected (Supplementary Fig. S4). The external menthol application methods were different in the studies: spray on the top wear^14,28–30^, whole-body creaming^13^ or immersion^18^, and gel on the face^25^ (Supplementary Table S4). The location of the administration and the surface area may also influence the effect since thermal signals from hairy skin provide more important feedback signals for the thermoregulation system than thermal signals from non-hairy skin; the latter functioning predominantly as an effector rather than a sensor^42^. To examine the possibility that treatment of a certain area of the body (e.g., face) with menthol has bigger impact on endurance performance than other areas in TTE protocols, we performed a sensitivity analysis (i.e., iteratively removing one study from the analyses and recalculating WMD to investigate the impact of each individual study on the summary estimate), which showed no difference in the final pooled results (Supplementary Table S5). Among the environmental factors, no meaningful difference was observed between subgroups with and without airflow in TT protocols (Supplementary Fig. S5a). In TTE tests, we found that menthol increased performance time when a fan (i.e., airflow) was present (WMD = 2.24 min; 95% CI 0.63 to 3.84 min) compared to no use of a fan (WMD = 0.64 min; 95% CI − 1.17 to 2.45 min) (Supplementary Fig. S5b). However, the averaged result of the subgroup with airflow should be taken with scrutiny due to the low number (n = 2) of studies in this subgroup. Furthermore, in TTE tests at higher T_a_s (above 31 °C) performance time was significantly increased in response to menthol compared with T_a_s ranging from ~ 20 to 30 °C (WMD = 1.02; 95% CI, − 0.01 to 2.04 min versus WMD = − 2.77 min; 95% CI, − 10.66 to 5.12 min; p < 0.001), while there was no significant difference between the subgroups in TT protocols (Supplementary Fig. S6).

## Discussion

In the present study, we show that the application of menthol improves TS, TC, and power output during physical exercise. Our results about thermal perception are in harmony with the findings of a previous meta-analysis^43^, which also showed beneficial effects of menthol on TS in exercise performance; however, in that study the thermophysiological effects of menthol were not analysed and influencing factors (e.g., acclimation, T_a_) of menthol’s effect were not investigated. In our study, we aimed at filling those gaps by studying the effects of menthol also on thermophysiological parameters, i.e., sweat production, heart rate, and deep T_b_, and by identifying different phenotypes and environmental factors which can augment or attenuate menthol’s effects. We show that the use of menthol does not lead to compromised warmth-defence responses during physical exercise, since it does not affect sweat production, heart rate, and deep T_b_. We also identify bodily (viz., higher BMI), methodological (i.e., external menthol administration), and environmental factors, such air movement (fan use) and higher T_a_, which enhance the beneficial effects of menthol on performance time.

Thermoregulatory changes, particularly in TS and TC, during physical exercise are of high importance, as they are considered among the limiting factors of endurance performance, and, as such, play a role in the development of fatigue^44,45^. The active muscle generates heat during physical exercise, thereby constituting an internal heat stress for the body, which is further augmented when physical activity is performed in the heat^46^. The heat load leads to worsening of TS and TC^44^, while behavioural and autonomic warmth-defence mechanisms are recruited to prevent an excessive increase in deep T_b_^46^. When the defence mechanisms are compromised or exhausted, bodily homeostasis cannot be maintained, and heat-related illnesses, such as exertional heat stroke in the most severe forms, develop^44^. Efforts should be made to prevent the simultaneous presence of severe external and internal heat load to the individuals. There are, however, certain scenarios, when prevention of these conditions is not possible. The most obvious examples include the strenuous physical activity of firefighters, soldiers, and professional athletes in hot environments.

As part of the global climate change, the incidence of heat waves has increased in different countries, including, for example, the UK^47^, France^48^, the US^49^, Australia^50^, and Japan^50^. These countries can be actual or potential hosts of upcoming worldwide, summertime sport events, e.g., Summer Olympic Games, thus pre-cautions should be implemented in order to prevent heat-related illnesses of the athletes during the games. In addition to physical methods of cooling, menthol may be also used as a pharmacological cooling intervention prior to and during exercise in hot conditions^50^. The improved TC in response to menthol can increase the thermal tolerance in athletes^51^, which can lead to better performance. It should be noted, however, that menthol may not be safely used to improve TC in athletes competing at cold environments (e.g., at T_a_ below 20 °C). Our analysis, to our knowledge for the first time, also shows that the application of menthol did not result in compromised warmth defences.

Menthol has been identified earlier as a cold-mimicking substance, and its beneficial effects on sport performance have been also reported in a recent review^43^. It is also known that menthol evokes its thermoregulatory effects through the TRPM8 channel, which, at least in rodents, serves as a universal cold receptor for the body^19^. The activation of TRPM8 (e.g., by cold or menthol) leads to the recruitment of cold-defence responses, which aim at elevating (but at least preventing the drop in) deep T_b_^11^. These thermophysiological effects of menthol, which were mostly discovered in animal experiments, imply a risk of menthol application in humans during physical exercise, since an adverse thermoregulatory effect, viz., an overt increase in deep T_b_, can not be ruled out. However, in humans thermal signals from the skin are less important for autonomic thermoregulation because the greater thermal inertia makes transient thermal exposures less threatening, thus decreases the importance of signals from the skin^42^. Hence, activating peripheral cold receptors, such as TRPM8, with menthol in humans can have smaller effects on T_b_ than in rodents. It should be also noted that signals used for behavioural thermoregulation, which can be triggered through altered TS or TC, can differ from signals for autonomic thermoregulation ^52^. For example, antagonists of the TRP vanilloid-1 channel readily affect autonomic thermoeffectors in rats^53^, but fail to affect the behavioural thermoeffectors in the same species, at least as concluded from one study^54^. Moreover, the mode of action for the thermal effect of TRP vanilloid-1 channel antagonists differs between rodents and humans^55^. Therefore, activation of peripheral thermosensation with menthol in humans can have smaller effects on deep T_b_ than in rodents.

In the present study, we collected the available information about the thermoregulation homeostasis in menthol-treated athletes performing exercise, and conducted meta-analysis of the obtained data. We showed that at the used doses, menthol exerted beneficial effects on endurance performance, but it had no significant effect on any of the thermoregulation-related parameters, which included sweating production, heart rate, and deep T_b_. It should be noted that sweat rate could be also an important indicator of thermoregulatory warmth defence. We found only three studies^14,20,25^, which reported sweat rate, but in all of them only the averaged sweat rate was reported for the treatment groups. In two studies^20,25^, there was no significant difference in sweat rate between menthol-treated and control groups, whereas in the third study the average sweat rate was significantly reduced after menthol treatment^14^. However, sweat rate is not steady, but rather a dynamic parameter during exercise. It was shown that during exercise sweating rate increased abruptly for 8 min after the onset of sweating and then continued increasing at a much lower rate^56^, therefore the average sweat rate for the entire duration of the exercise should be interpreted with caution. As an alternative, we compared the exercise durations between the menthol-treated and control groups of the studies that reported sweat production and found that the difference between the treatment groups was less than 3 min in all studies^14,17,28–30^. We believe that such minimal difference in exercise duration between treatment groups of the same study did not have a significant influence on sweat production. Our results suggest that with regards to thermoregulation homeostasis, menthol can be safely applied during physical exercise in humans. Nevertheless, it is also possible that the administered doses of menthol and the treated surface area were not sufficient in the most of the analysed studies to trigger cold-defence responses, thereby leading to a change in thermophysiological parameters, including deep T_b_. Furthermore, we pointed out different influencing factors, which can help to augment the performance-improving effects of menthol. Among environmental factors, we found that the use of a fan (i.e., wind effect) and higher T_a_ increased the efficacy of menthol on endurance performance. The beneficial effects of menthol were more pronounced in subjects with higher BMI, while acclimation to heat did not influence the effects.

Some limitations of our study should be also mentioned. There were inter-study differences in the design of the analysed studies regarding, for example, the sample population, the menthol administration route and dose, the exercise protocol, and the measurement of the outcome parameters. For example, the assessment of power output differed in the three analysed studies^18,28,30^. The study with the biggest effect size showed a significant improvement in power output^18^, whereas power output did not differ statistically between the menthol-treated and control groups in either of the studies with smaller effect size^57,58^. Based on the risk of bias assessment, we found that blinding was not feasible in many studies, because of the characteristic odour of menthol. Furthermore, the allocation concealment was not indicated in some articles^13,17,18,20,24,25,31^, which could have also influenced the effectiveness of menthol application. These methodological and medical differences in study design can explain the considerably high between-study heterogeneity (indicated by an I^2^ of more than 50%), as observed in our analysis (Figs. 2 and 8b; Supplementary Figs. S1-S6). To account for the presence of heterogeneity, we used the random effects model in all forest plots of our meta-analyses. However, it is still possible that, despite all of our approaches to reduce methodological errors, the high heterogeneity of the analysed studies might have negatively impacted our results.

Our findings suggest that menthol can be safely used during physical exercise to improve thermal perception. Due to its beneficial effects on TS and TC, it can be used as an alternative to mitigate the impact of heat exposure on the individuals. External application of menthol in a warmer environment with air movement is more efficient, especially in subjects with higher BMI than 23. The validation of our results in targeted human trials is subject for future research.

## Supplementary information

Supplementary Information.

## Data Availability

All data generated or analysed during this study are included in this published article.
