# Supplementary material for: Menthol can be safely applied to improve thermal perception during physical exercise: a meta-analysis of randomized controlled trials
Source: Sci Rep. 2020 Aug 12;10:13636. doi: 10.1038/s41598-020-70499-9 (PMC7423903; doi:10.1038/s41598-020-70499-9)
Supplement: Supplementary file 1 — Supplementary Information. [file 41598_2020_70499_MOESM1_ESM.docx]

**Supplementary Information**

**Menthol can be safely applied to improve thermal perception during physical exercise: a meta-analysis of randomized controlled trials**

Patrik Keringer, Nelli Farkas, Noemi Gede, Peter Hegyi, Zoltan Rumbus, Zsolt Lohinai, Margit Solymar, Kasidid Ruksakiet, Gabor Varga, Andras Garami

**Table of contents:**

**Tables:**

**Table S1.** PRISMA checklist.

**Table S2.** Risk of bias assessment using the Cochrane Risk of Bias Tool for Randomized Controlled Trials.

**Table S3.** Characteristics of participants in the studies included in the meta-analysis.

**Table S4.** Menthol application methods, exercise protocols, and environmental conditions in the studies eligible for quantitative analysis.

**Table S5.** Sensitivity analysis for performance time showing the effect of menthol in time-to-exhaustion tests in different external menthol application methods (spray on the top wear [Barwood 2019], cream over the whole body [Kounalakis 2010] and gel on the face [Schlader 2011]).

**Figures:**

**Figure S1.** Forest plot of the weighted mean differences (WMDs) for thermal sensation showing the effect of menthol in subgroups with (top) and without (bottom) airflow.

**Figure S2.** Forest plot of the weighted mean of differences (WMDs) for performance time in (**a**) time-trial (TT) and (**b**) time-to-exhaustion (TTE) tests showing the effect of menthol during exercise.

**Figure S3.** Forest plot of the weighted mean differences (WMDs) for performance time showing the effect of menthol in (**a**) time-trial (TT) and (**b**) time-to-exhaustion (TTE) tests in subgroups of non-acclimated and acclimated participants.

**Figure S4.** Forest plot of the weighted mean differences (WMDs) for performance time showing the effect of menthol in (**a**) time-trial (TT) and (**b**) time-to-exhaustion (TTE) tests in subgroups of external and internal application.

**Figure S5.** Forest plot of the weighted mean differences (WMDs) for performance time showing the effect of menthol in (**a**) time-trial (TT) and (**b**) time-to-exhaustion (TTE) tests in subgroups without and with airflow.

**Figure S6.** Forest plot of the weighted mean differences (WMDs) for performance time showing the effect of menthol in (**a**) time-trial (TT) and (**b**) time-to-exhaustion (TTE) tests in subgroups of lower (< 31°C) and higher (31°C and above) ambient temperature (T_a_).

**Figure S7.** Funnel plot of the studies that were included in the forest plot of menthol’s effect on thermal sensation (n = 9, Egger’s test: p = 0.073). Here, and in Figures S8-S10, the dots represent results from studies included in the forest plot.

**Figure S8.** Funnel plot of the studies that were included in the forest plot of menthol’s effect on core temperature (n = 10, Egger’s test: p = 0.341).

**Figure S9.** Funnel plot of the studies that were included in the forest plot of menthol’s effect on heart rate (n = 8, Egger’s test: p = 0.064).

**Figure S10.** Funnel plot of the studies that were included in the forest plot of menthol’s effect on performance time in time-to-exhaustion exercise protocols (n = 9, Egger’s test: p = 0.877).

**Table S1.** PRISMA checklist**.**

| **Section/topic** | **#** | **Checklist item** | **Reported on page #** |
| --- | --- | --- | --- |
| **TITLE** | | |  |
| Title | 1 | Identify the report as a systematic review, meta-analysis, or both. | 1 |
| **ABSTRACT** | | |  |
| Structured summary | 2 | Provide a structured summary including, as applicable: background; objectives; data sources; study eligibility criteria, participants, and interventions; study appraisal and synthesis methods; results; limitations; conclusions and implications of key findings; systematic review registration number. | 1 |
| **INTRODUCTION** | | |  |
| Rationale | 3 | Describe the rationale for the review in the context of what is already known. | 1-2 |
| Objectives | 4 | Provide an explicit statement of questions being addressed with reference to participants, interventions, comparisons, outcomes, and study design (PICOS). | 2 |
| **METHODS** | | |  |
| Protocol and registration | 5 | Indicate if a review protocol exists, if and where it can be accessed (e.g., Web address), and, if available, provide registration information including registration number. | 2 |
| Eligibility criteria | 6 | Specify study characteristics (e.g., PICOS, length of follow-up) and report characteristics (e.g., years considered, language, publication status) used as criteria for eligibility, giving rationale. | 2 |
| Information sources | 7 | Describe all information sources (e.g., databases with dates of coverage, contact with study authors to identify additional studies) in the search and date last searched. | 2 |
| Search | 8 | Present full electronic search strategy for at least one database, including any limits used, such that it could be repeated. | 2 |
| Study selection | 9 | State the process for selecting studies (i.e., screening, eligibility, included in systematic review, and, if applicable, included in the meta-analysis). | 2 |
| Data collection process | 10 | Describe method of data extraction from reports (e.g., piloted forms, independently, in duplicate) and any processes for obtaining and confirming data from investigators. | 2 |
| Data items | 11 | List and define all variables for which data were sought (e.g., PICOS, funding sources) and any assumptions and simplifications made. | 2 |
| Risk of bias in individual studies | 12 | Describe methods used for assessing risk of bias of individual studies (including specification of whether this was done at the study or outcome level), and how this information is to be used in any data synthesis. | 2 |
| Summary measures | 13 | State the principal summary measures (e.g., risk ratio, difference in means). | 2 |

| Synthesis of results | 14 | Describe the methods of handling data and combining results of studies, if done, including measures of consistency (e.g., I^2^) for each meta-analysis. | 2-3 |
| --- | --- | --- | --- |
| Risk of bias across studies | 15 | Specify any assessment of risk of bias that may affect the cumulative evidence (e.g., publication bias, selective reporting within studies). | 2-3 |
| Additional analyses | 16 | Describe methods of additional analyses (e.g., sensitivity or subgroup analyses, meta-regression), if done, indicating which were pre-specified. | 2-3 |
| **RESULTS** | | |  |
| Study selection | 17 | Give numbers of studies screened, assessed for eligibility, and included in the review, with reasons for exclusions at each stage, ideally with a flow diagram. | 3; Fig. 1 |
| Study characteristics | 18 | For each study, present characteristics for which data were extracted (e.g., study size, PICOS, follow-up period) and provide the citations. | 3; Tables S3-S4 |
| Risk of bias within studies | 19 | Present data on risk of bias of each study and, if available, any outcome level assessment (see item 12). | 3; Table S2 |
| Results of individual studies | 20 | For all outcomes considered (benefits or harms), present, for each study: (a) simple summary data for each intervention group (b) effect estimates and confidence intervals, ideally with a forest plot. | 4-8;  Figures 2-8; Figures S1-S6 |
| Synthesis of results | 21 | Present results of each meta-analysis done, including confidence intervals and measures of consistency. | 4-8; Figures 2-8; Figures S1-S6 |
| Risk of bias across studies | 22 | Present results of any assessment of risk of bias across studies (see Item 15). | 4-8; Table S2 |
| Additional analysis | 23 | Give results of additional analyses, if done (e.g., sensitivity or subgroup analyses, meta-regression [see Item 16]). | 4-8; Table S5 |
| **DISCUSSION** | | |  |
| Summary of evidence | 24 | Summarize the main findings including the strength of evidence for each main outcome; consider their relevance to key groups (e.g., healthcare providers, users, and policy makers). | 8-10 |
| Limitations | 25 | Discuss limitations at study and outcome level (e.g., risk of bias), and at review-level (e.g., incomplete retrieval of identified research, reporting bias). | 10 |
| Conclusions | 26 | Provide a general interpretation of the results in the context of other evidence, and implications for future research. | 10 |
| **FUNDING** | | |  |
| Funding | 27 | Describe sources of funding for the systematic review and other support (e.g., supply of data); role of funders for the systematic review. | 12 |

**Table S2.** Risk of bias assessment using the Cochrane Risk of Bias Tool for Randomized Controlled Trials.


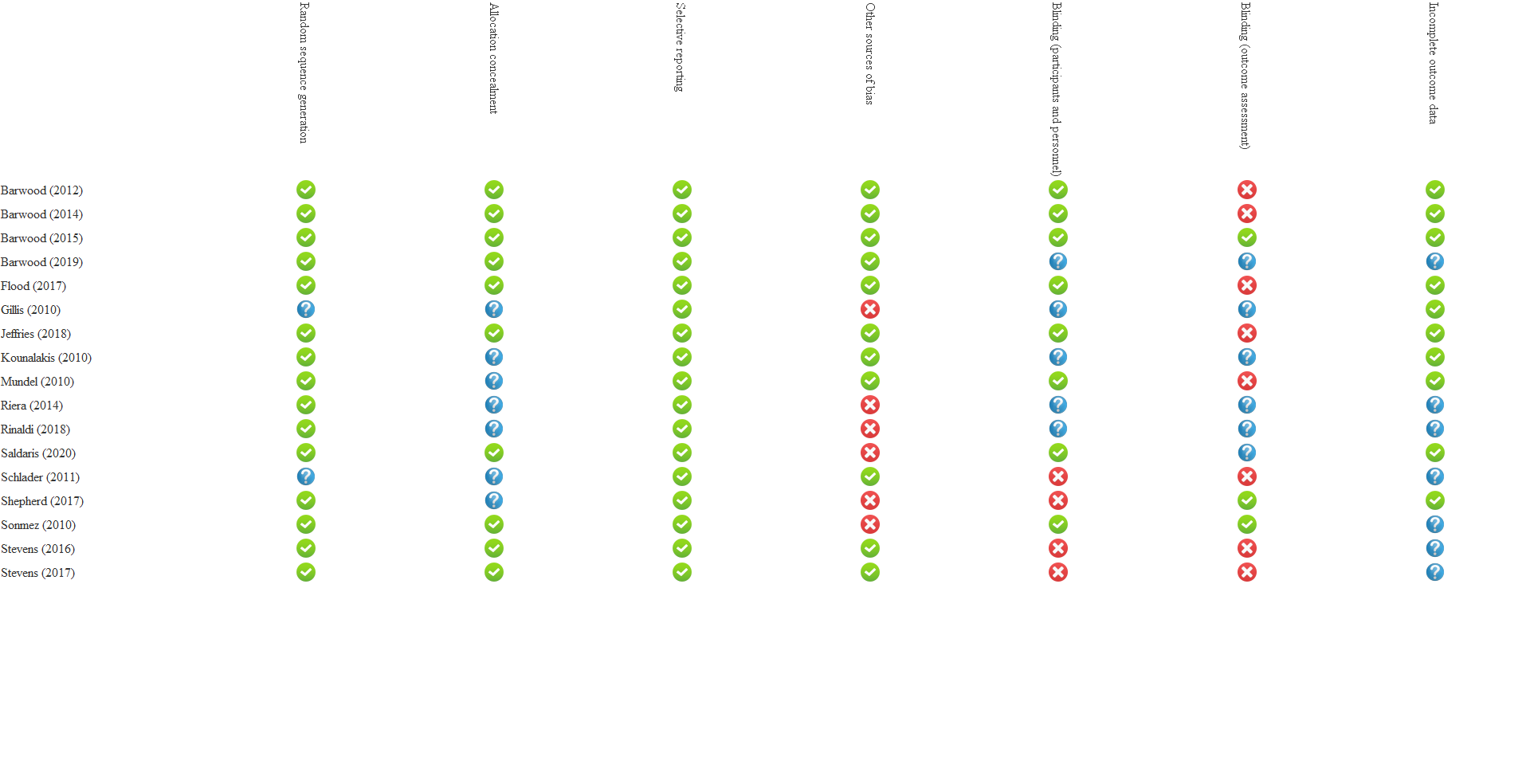


According to the Cochrane Risk of Bias Tool, 7 studies are considered overall as poor quality (Gillis 2010, Riera 2014, Rinaldi 2018, Schlader 2011, Shepherd 2017, Stevens 2016, Stevens 2017), as two of the points were determined as “potentially high risk of bias”; 9 studies as moderate quality (Barwood 2012, Barwood 2014, Barwood 2019, Flood 2017, Jeffries 2018, Kounalakis 2010, Mundel 2010, Saldaris 2020, Sonmez 2010), since one criterion was determined as “potentially high risk of bias” or two criteria as “unclear risk of bias”; and one study as high quality (Barwood 2015), for every points were determined as “low risk of bias”.


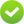
, low risk; , high risk; , unclear risk of bias.


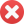

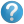


**Table S3.** Characteristics of participants in the studies included in the meta-analysis.

| Study | Number of participants | Mean age  (years) | Mean BMI (kg/m^2^) | Trained? | Acclimated? |
| --- | --- | --- | --- | --- | --- |
| Barwood (2012) | 11 | 30 | 24.0 | Yes | No |
| Barwood (2014) | 6 | 21 | 24.4 | Yes | NR |
| Barwood (2015) | 8 | 21 | 25.4 | Yes | NR |
| Barwood (2019) | 8 | 22 | NR | Yes | NR |
| Flood (2017) | 8 | 26 | 24.2 | NR | No |
| Gillis (2010) | 12 | 22 | 23.6 | Yes | NR |
| Jeffries (2018) | 10 | 33 | 23.7 | NR | No |
| Kounalakis (2010) | 8  8 | 28  21 | 22.2  23.2 | Yes  Yes | NR  NR |
| Mundel (2010) | 9 | 25 | 25.0 | NR | No |
| Riera (2014) | 12 | 42 | 22.8 | Yes | Yes |
| Rinaldi (2018) | 8 | 24 | NR | Yes | Yes |
| Saldaris (2020) | 12 | 25 | 23.4 | Yes | No |
| Schlader (2011) | 12 | 23 | 25.6 | Yes | NR |
| Shepherd (2017) | 7 | 25 | 24.3 | Yes | NR |
| Sonmez (2010) | 16 | 22 | NR | Yes | NR |
| Stevens (2016) | 11 | 29 | 23.4 | Yes | Yes |
| Stevens (2017) | 11 | 30 | 23.4 | Yes | Yes |

BMI, body mass index; NR, not reported

**Table S4.** Menthol application methods, exercise protocols, and environmental conditions in the studies eligible for quantitative analysis.

| First author (publication year) | Menthol concentration (%); volume (ml) | Administration method; body surface area (%)^a^ | Warm up | Exercise protocol; approx. duration (min) | T_a_ (°C)^b^ | Relative humidity (%)^b^ |
| --- | --- | --- | --- | --- | --- | --- |
| Barwood (2012) | 0.05; 100 | External (spray on top wear); 36 | Yes | Cycling TT; 71 | 31.5 (0.7) | 53 (5) |
| Barwood (2014) | 0.2; 100 | External (spray on top wear); 36 | Yes | Running TT; 28 | 33.9 (0.1) | 55 |
| Barwood (2015) | 0.2; 100 | External (spray on top wear); 36 | Yes | Cycling TT; 33 | 33.5 (0.5) | 33 (5) |
| Barwood (2019) | 0.2; 100 | External (spray on top wear); 36 | Yes | Cycling TTE; 49 | 34.6 (1.2) | 22 (1) |
| Flood (2017) | 0.01; 25 | Internal | Yes | Cycling TTE; 23 | 35.0 (0.8) | 48 (2) |
| Gillis (2010) | 0.05; 100 | External (spray on top wear); 36 | No | Cycling FTE; 45 | 26.3 (0.9) | 72 (3) |
| Jeffries (2018) | 0.01; 25 | Internal | Yes | Cycling TTE; 25 | 35.0 (0.2) | 40 (1) |
| Kounalakis (2010) | ^c^; 100 | External (cream on whole body); 91 | No | Cycling TTE; 27 | 24.1 (1) | 46 (4) |
| Mundel (2010) | 0.01; 25 | Internal | No | Cycling TTE; 61 | 34.0 (1) | 27 (4) |
| Riera (2014) | 0.01; 190 | Internal | Yes | Cycling TT; 37 | 30.7 (0.8) | 78 (0) |
| Rinaldi (2018) | 0.1; NR | External (whole-body immersion); 91 | Yes | Cycling FTE; 2 x 20 | 29.1 (1.5) | 62 (4) |
| Saldaris (2020) | 0.1; 25 | Internal | No | Running TTE; 1 | 35.3 (0.3) | 59 (3) |
| Schlader (2011) | 8; ^d^ | External (gel on face); 4 | Yes | Cycling TTE; 21 | 20.3 (0.2) | 48 (3) |
| Shepherd (2017) | 0.01; 500 | Internal | No | Cycling TTE; 10 | NR | NR |
| Sonmez (2010) | NR | Internal | No | Running TT; 1 | NR | NR |
| Stevens (2016) | 0.01; 25 | Internal | Yes | Running TT; 26 | 32.6 (0.2) | 46 (6) |
| Stevens (2017) | 0.01; 25 | Internal | No | Running TT; 14 | 32.5 (0.1) | 47 (8) |

^a^according to the Wallace rules of nine (Wallace, A. B. The exposure treatment of burns. *Lancet*, **257**, 501–504 [1951]), by omitting the area of the head from whole-body and of the arms from top wear applications; ^b^data are shown as mean (standard deviation); menthol was applied at ^c^4.6 g and ^d^0.5 g/100cm^2^; FTE, fixed-time exercise; NR, not reported; T_a_, ambient temperature; TT, time trial; TTE, time to exhaustion

**Table S5.** Sensitivity analysis for performance time showing the effect of menthol in time-to-exhaustion tests in different external menthol application methods (spray on the top wear [Barwood 2019], cream over the whole body [Kounalakis 2010] and gel on the face [Schlader 2011]).

| Study omitted | Estimate | 95% confidence interval | |
| --- | --- | --- | --- |
|  |  |  |  |
| Kounalakis (2010)/2 | 1.730273 | -0.61056 | 4.071108 |
| Kounalakis (2010)/1 | 2.278419 | 0.103842 | 4.452996 |
| Barwood (2019) | -2.76844 | -10.6567 | 5.119824 |
| Schlader (2011) | -2.9609 | -10.0378 | 4.116044 |
|  |  |  |  |
| Combined | 0.825719 | -1.94855 | 3.599985 |

(2019)

Menthol-control WMD in thermal sensation

Menthol Control

WMD (95% CI) N, mean (SD) N, mean (SD) Weight (%)

without airflow

with airflow

with airflow


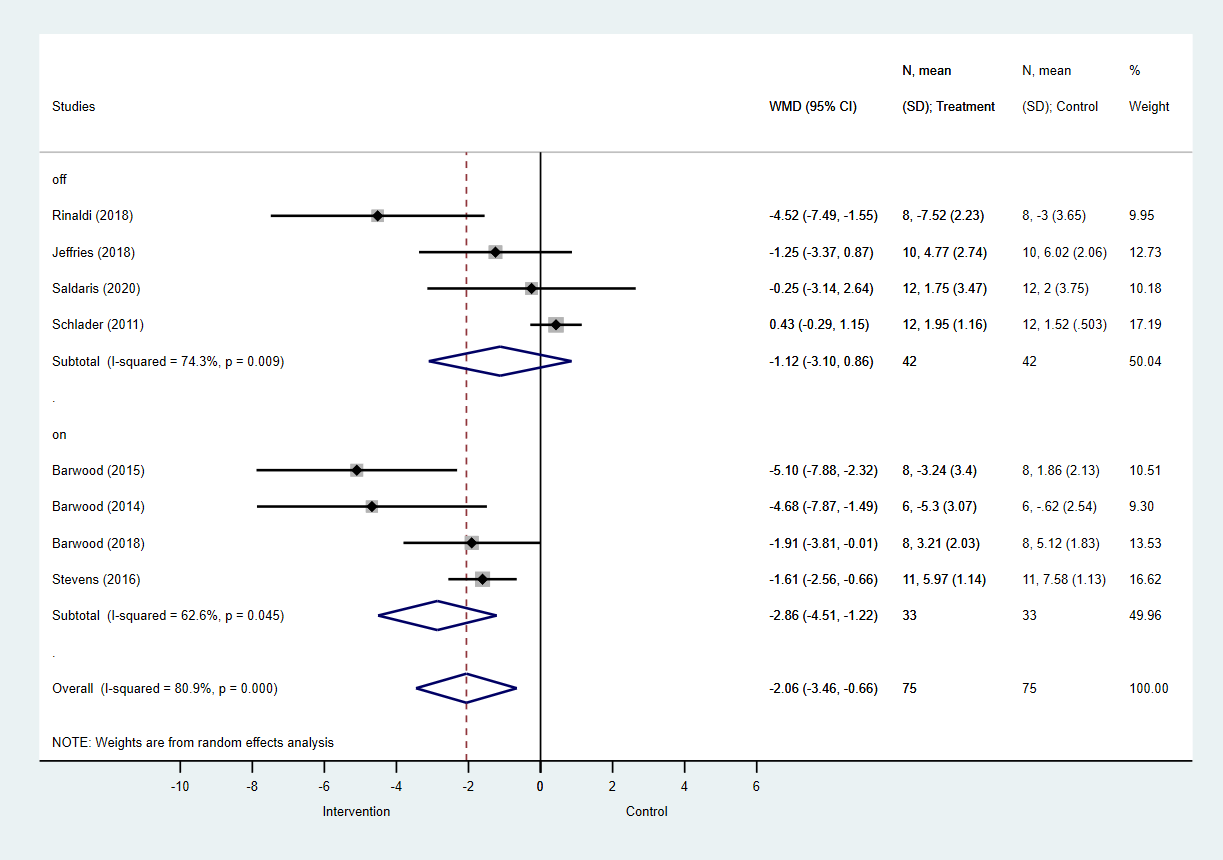


**Figure S1.** Forest plot of the weighted mean differences (WMDs) for thermal sensation showing the effect of menthol in subgroups with (top) and without (bottom) airflow.

Menthol Control

WMD (95% CI) N, mean (SD) N, mean (SD) Weight (%)

**a**


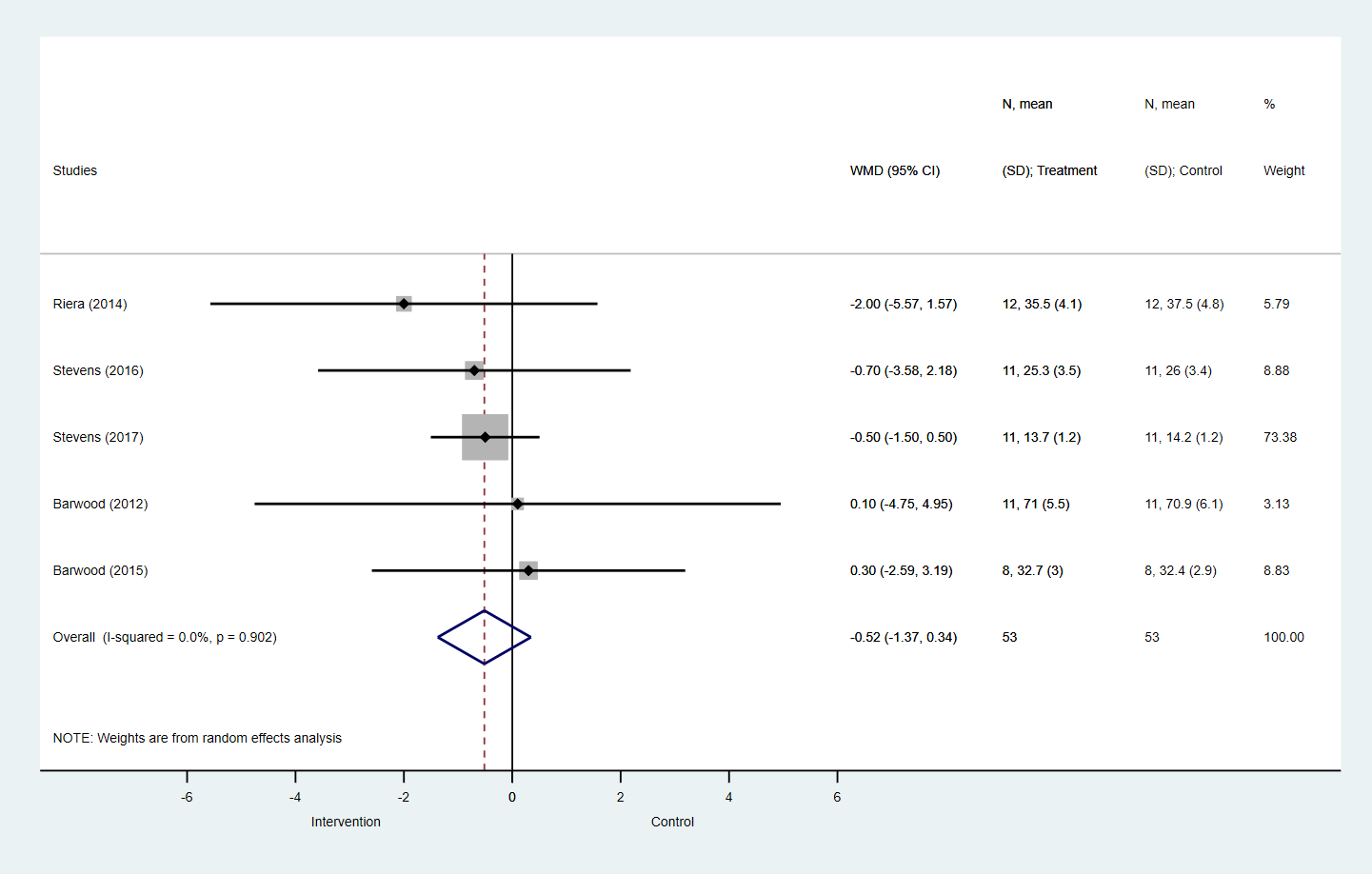


TT
Studies

Menthol-control WMD in performance time (min)


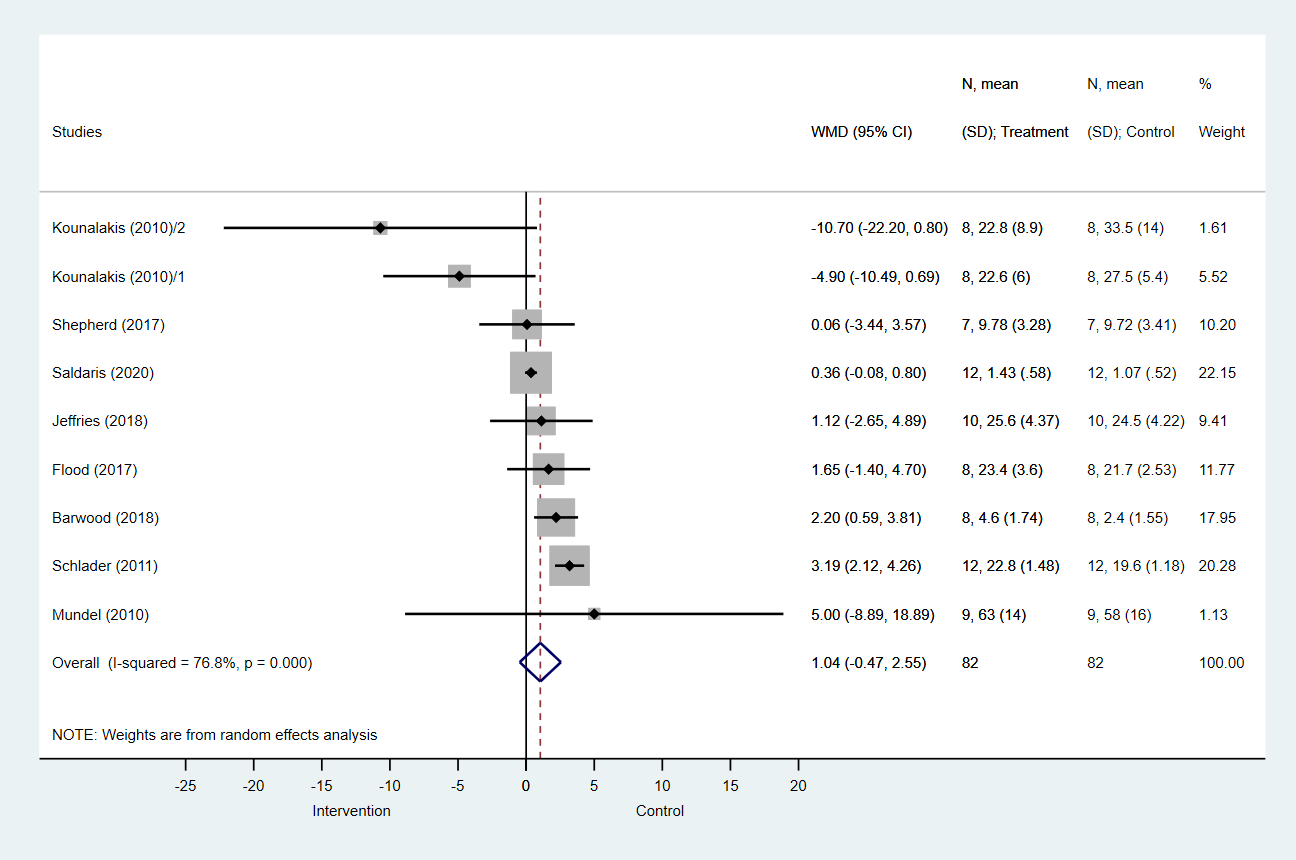


Menthol Control

WMD (95% CI) N, mean (SD) N, mean (SD) Weight (%)

**b**

TTE
Studies

(2019)

Menthol-control WMD in performance time (min)

**Figure S2.** Forest plot of the weighted mean of differences (WMDs) for performance time in (**a**) time-trial (TT) and (**b**) time-to-exhaustion (TTE) tests showing the effect of menthol during exercise.

Menthol Control

WMD (95% CI) N, mean (SD) N, mean (SD) Weight (%)

TT
Studies

**a**


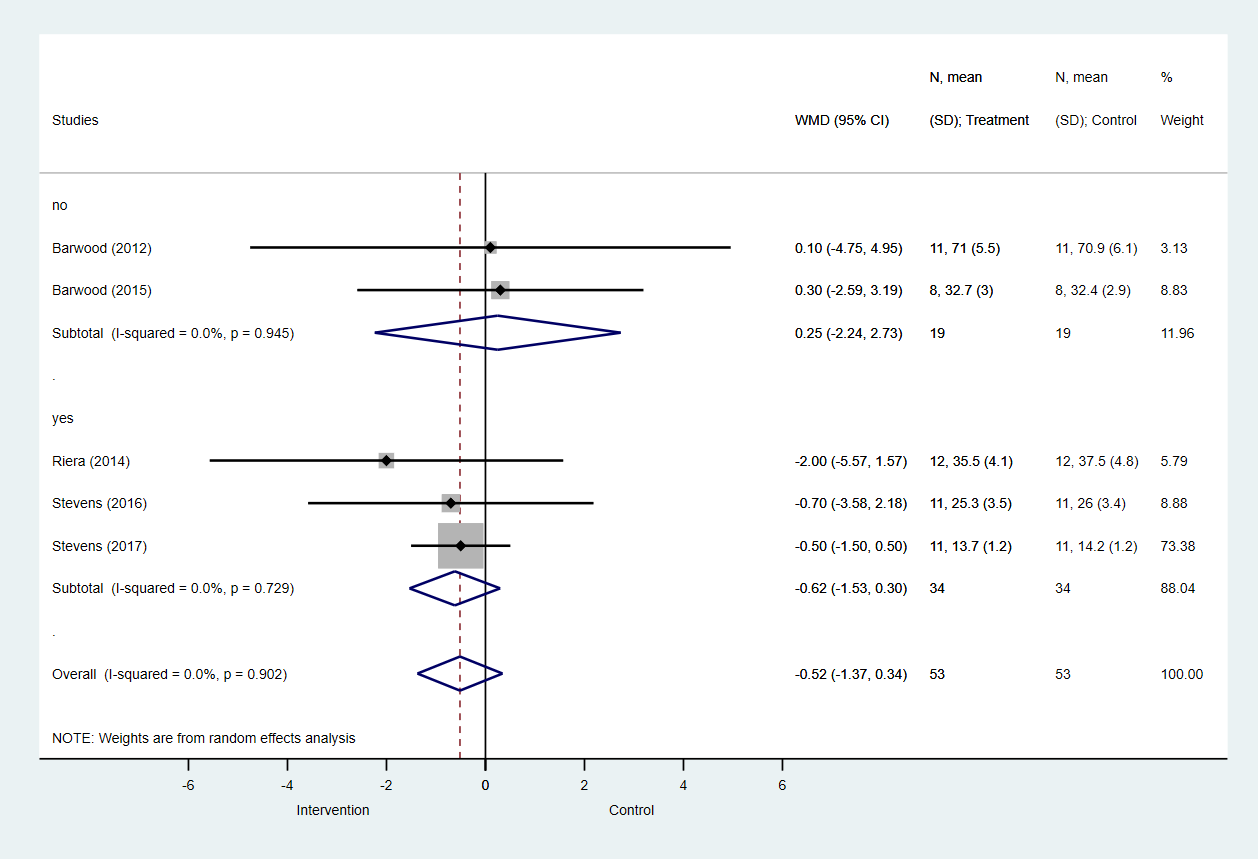


non-acclimated

acclimated

Menthol-control WMD in performance time (min)


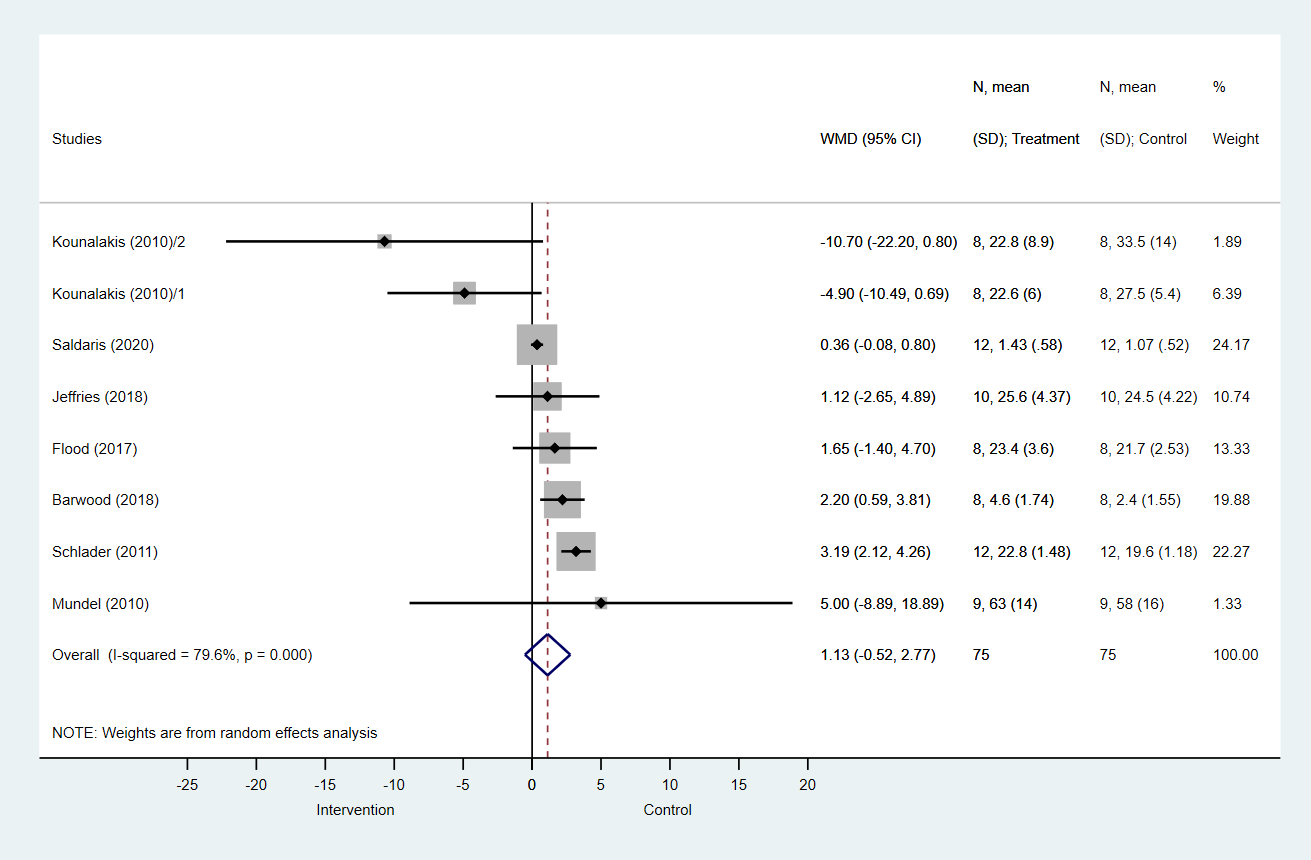


**b**

Menthol Control

WMD (95% CI) N, mean (SD) N, mean (SD) Weight (%)

TTE
Studies

non-acclimated

(2019)

Menthol-control WMD in performance time (min)

**Figure S3.** Forest plot of the weighted mean differences (WMDs) for performance time showing the effect of menthol in (**a**) time-trial (TT) and (**b**) time-to-exhaustion (TTE) tests in subgroups of non-acclimated and acclimated participants.

TT
Studies


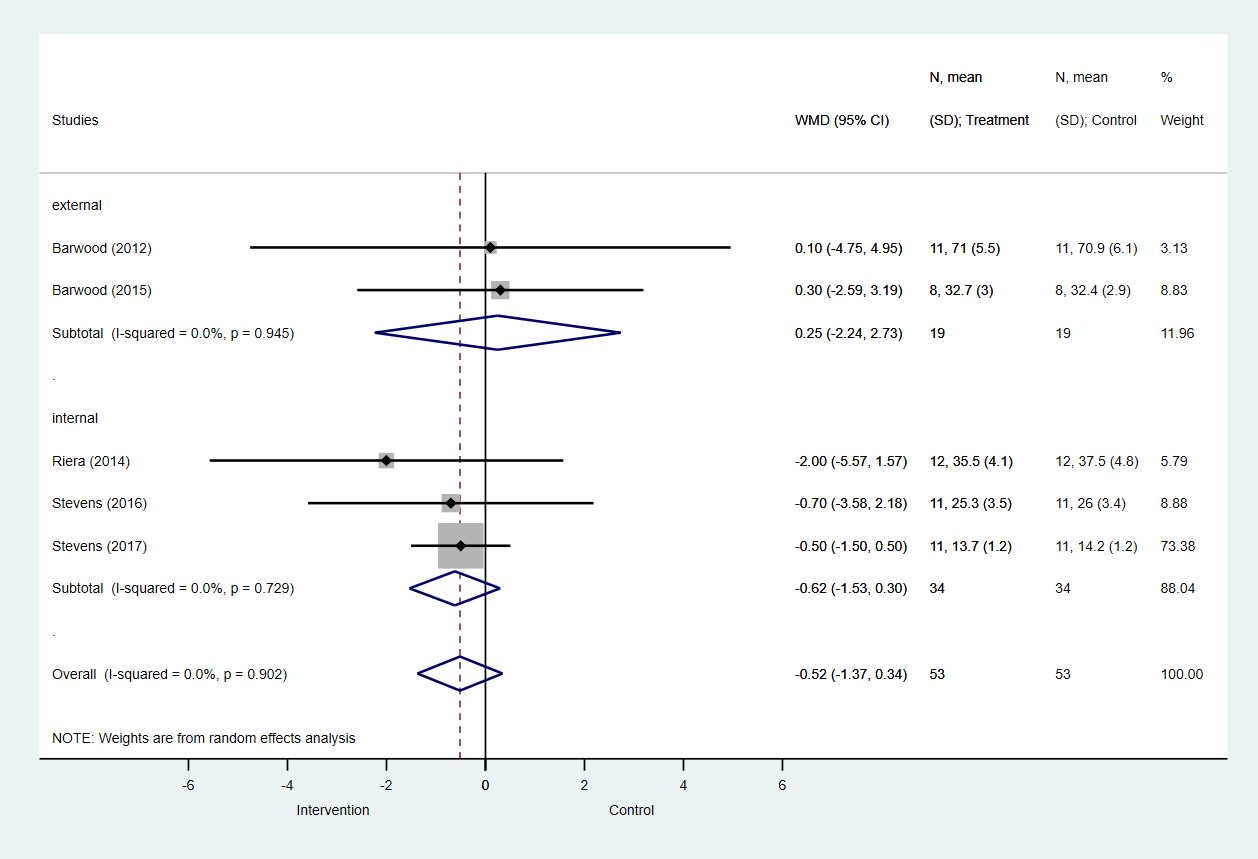


Menthol Control

WMD (95% CI) N, mean (SD) N, mean (SD) Weight (%)

**a**

Menthol-control WMD in performance time (min)

**b**


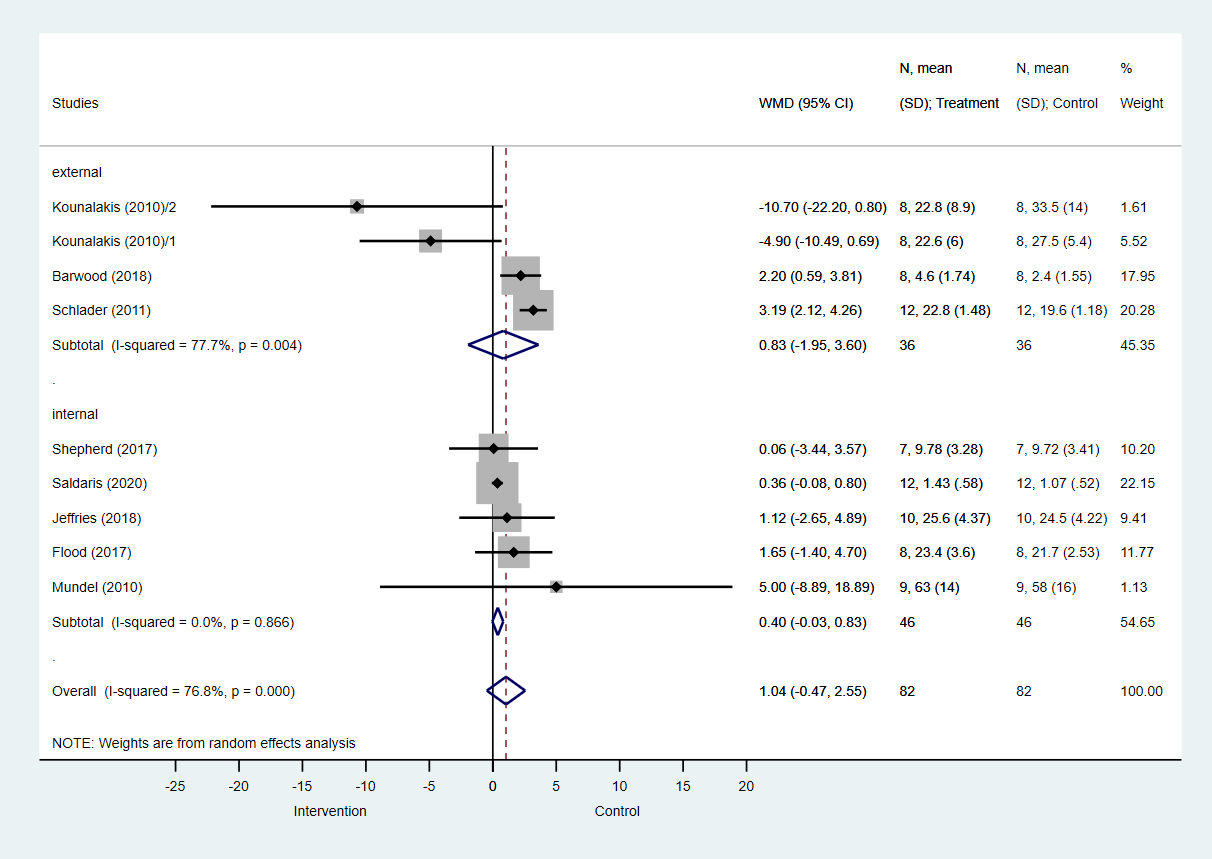


TTE
Studies

Menthol Control

WMD (95% CI) N, mean (SD) N, mean (SD) Weight (%)

(2019)

Menthol-control WMD in performance time (min)

**Figure S4.** Forest plot of the weighted mean differences (WMDs) for performance time showing the effect of menthol in (**a**) time-trial (TT) and (**b**) time-to-exhaustion (TTE) tests in subgroups of external and internal application.

Menthol Control

WMD (95% CI) N, mean (SD) N, mean (SD) Weight (%)

**a**


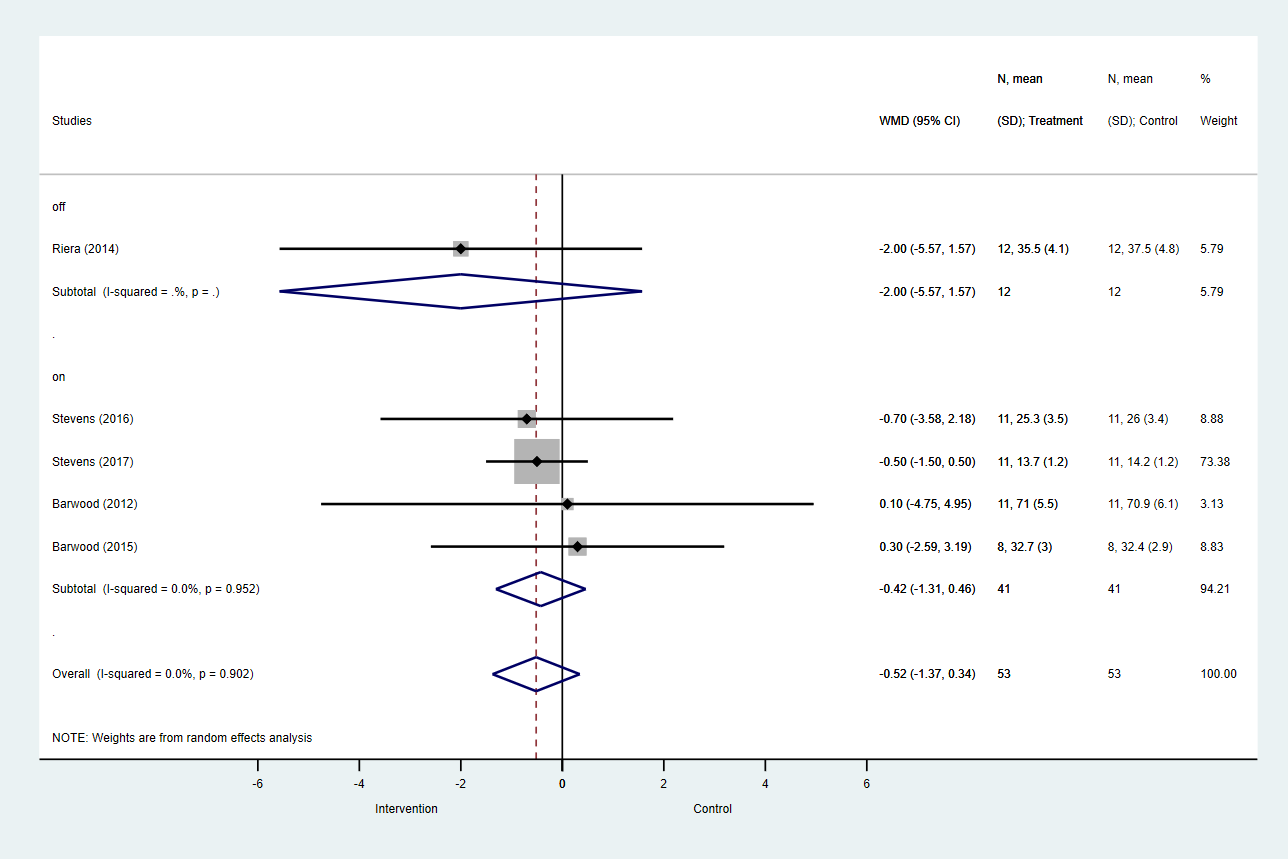


TT
Studies

without airflow

with airflow

Menthol-control WMD in performance time (min)

**b**


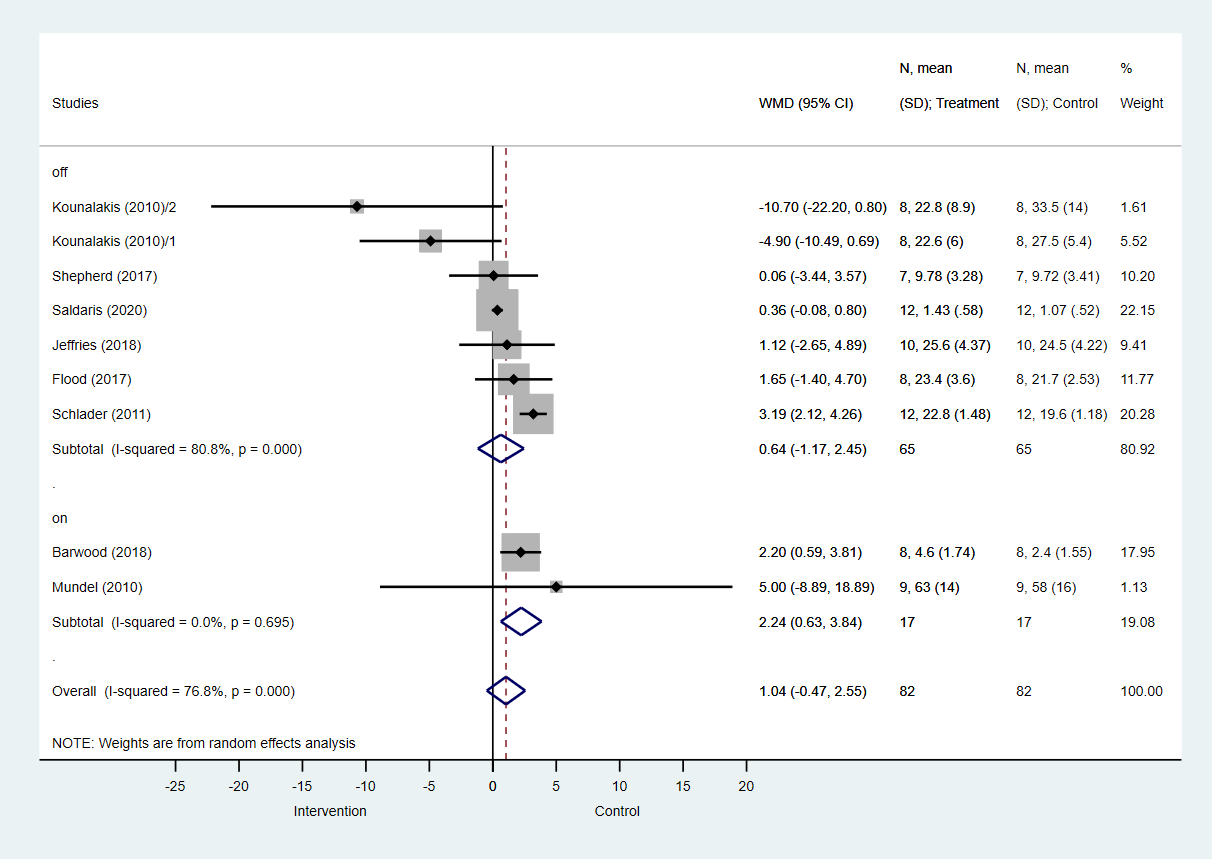


TTE
Studies

Menthol Control

WMD (95% CI) N, mean (SD) N, mean (SD) Weight (%)

without airflow

with airflow

(2019)

Menthol-control WMD in performance time (min)

**Figure S5.** Forest plot of the weighted mean differences (WMDs) for performance time showing the effect of menthol in (**a**) time-trial (TT) and (**b**) time-to-exhaustion (TTE) tests in subgroups without and with airflow.

Menthol Control

WMD (95% CI) N, mean (SD) N, mean (SD) Weight (%)

**a**


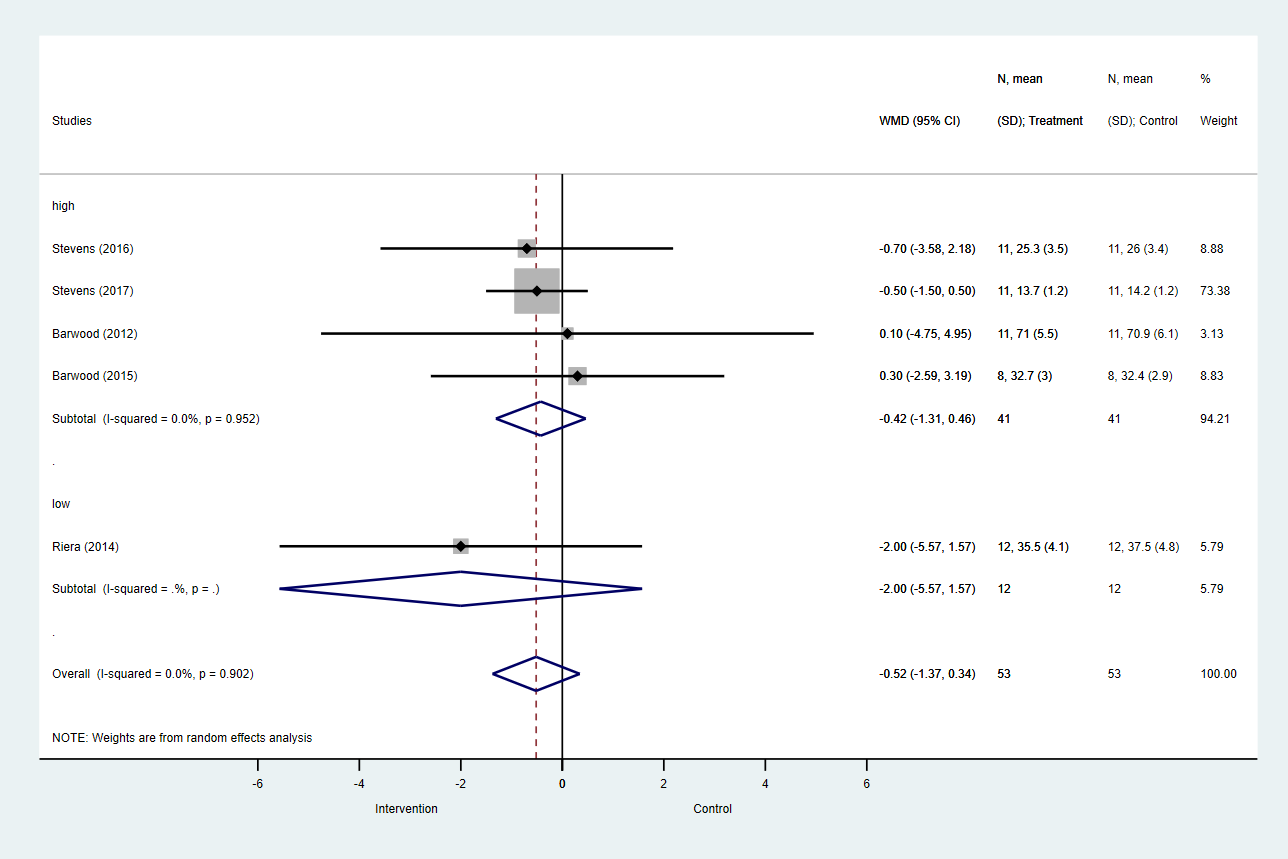


TT
Studies

**
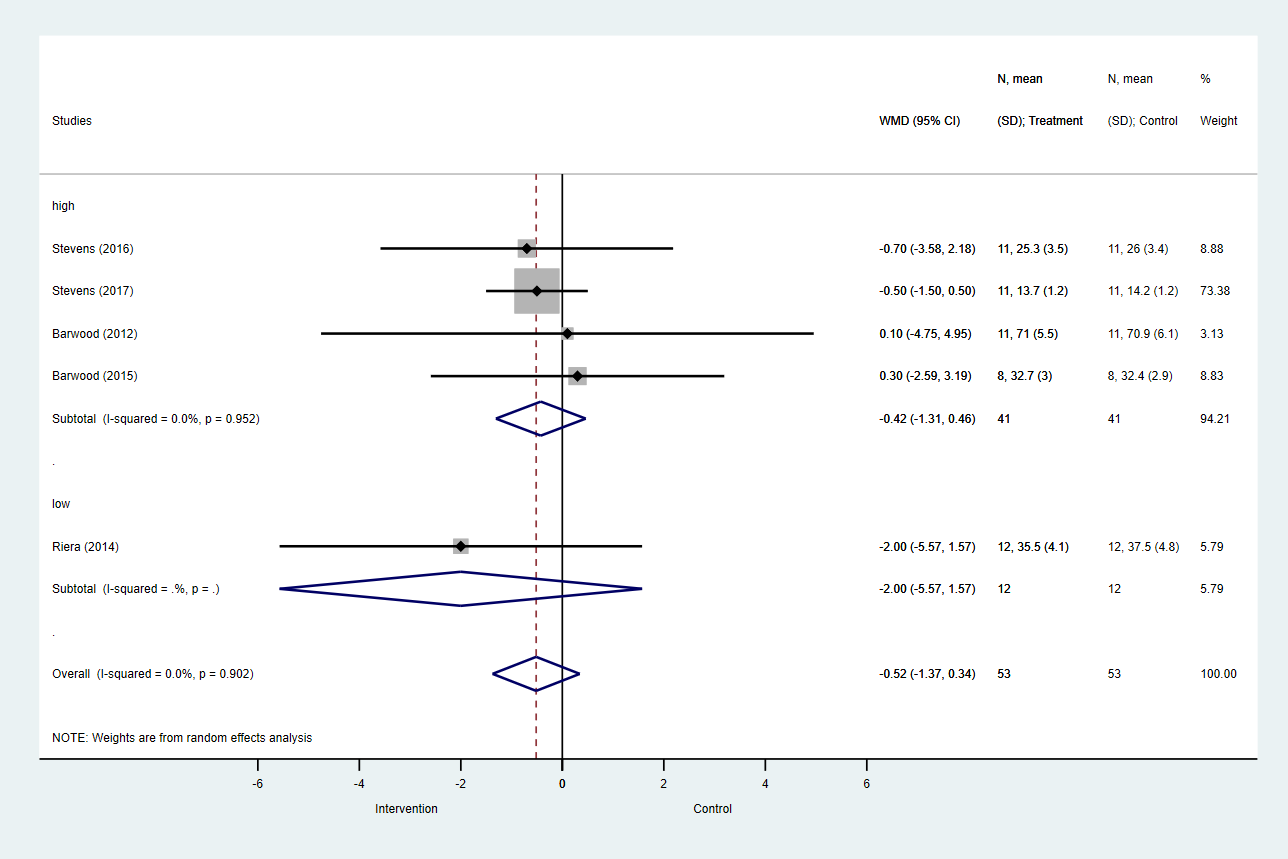
**

lower T_a_


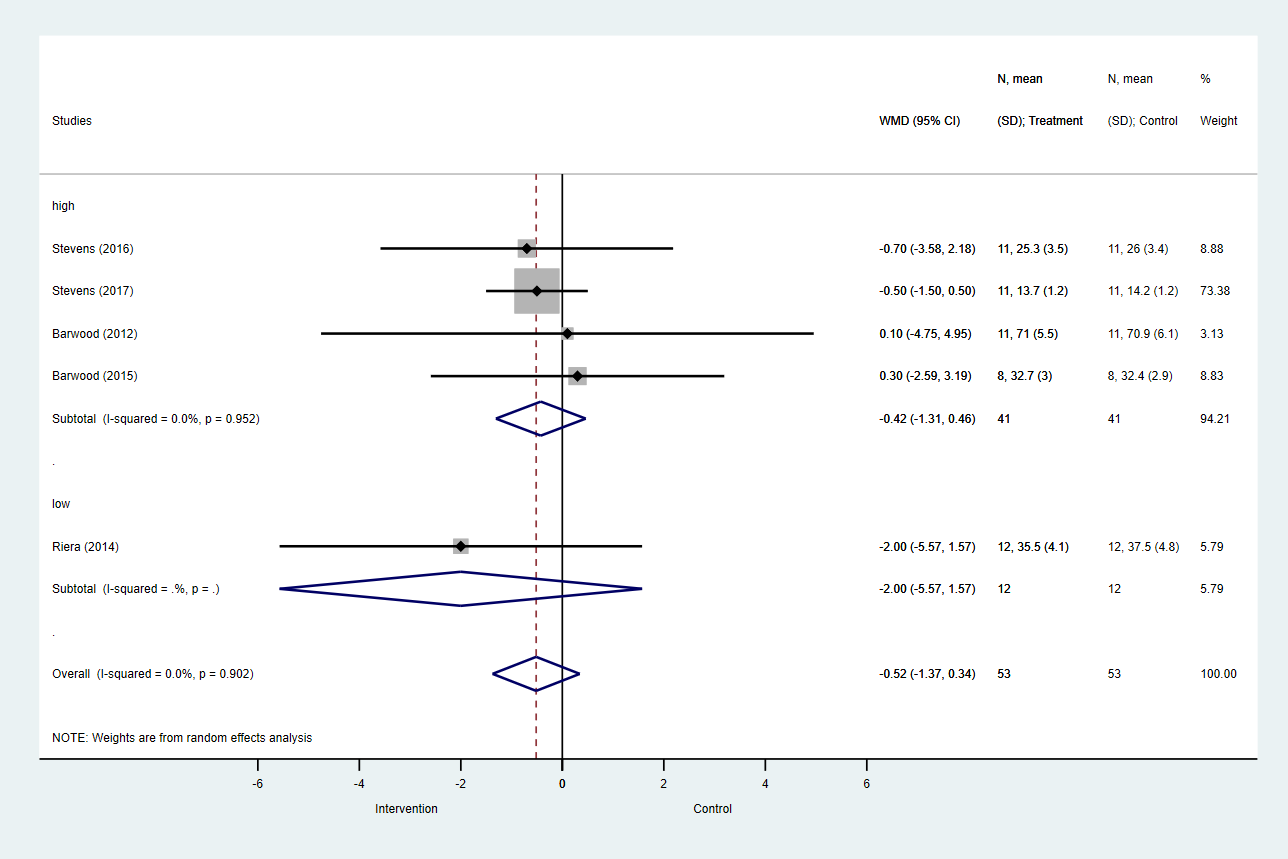


higher T_a_

**
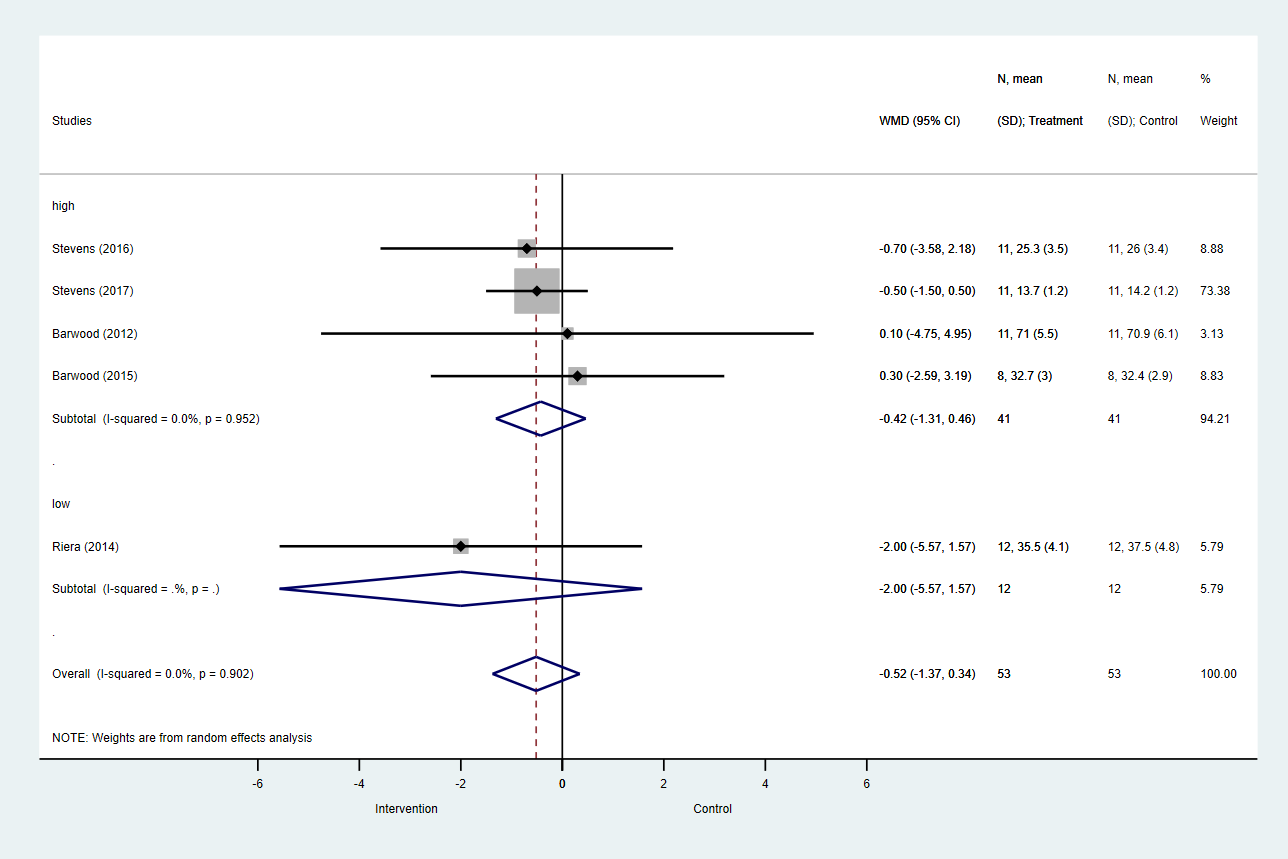
**


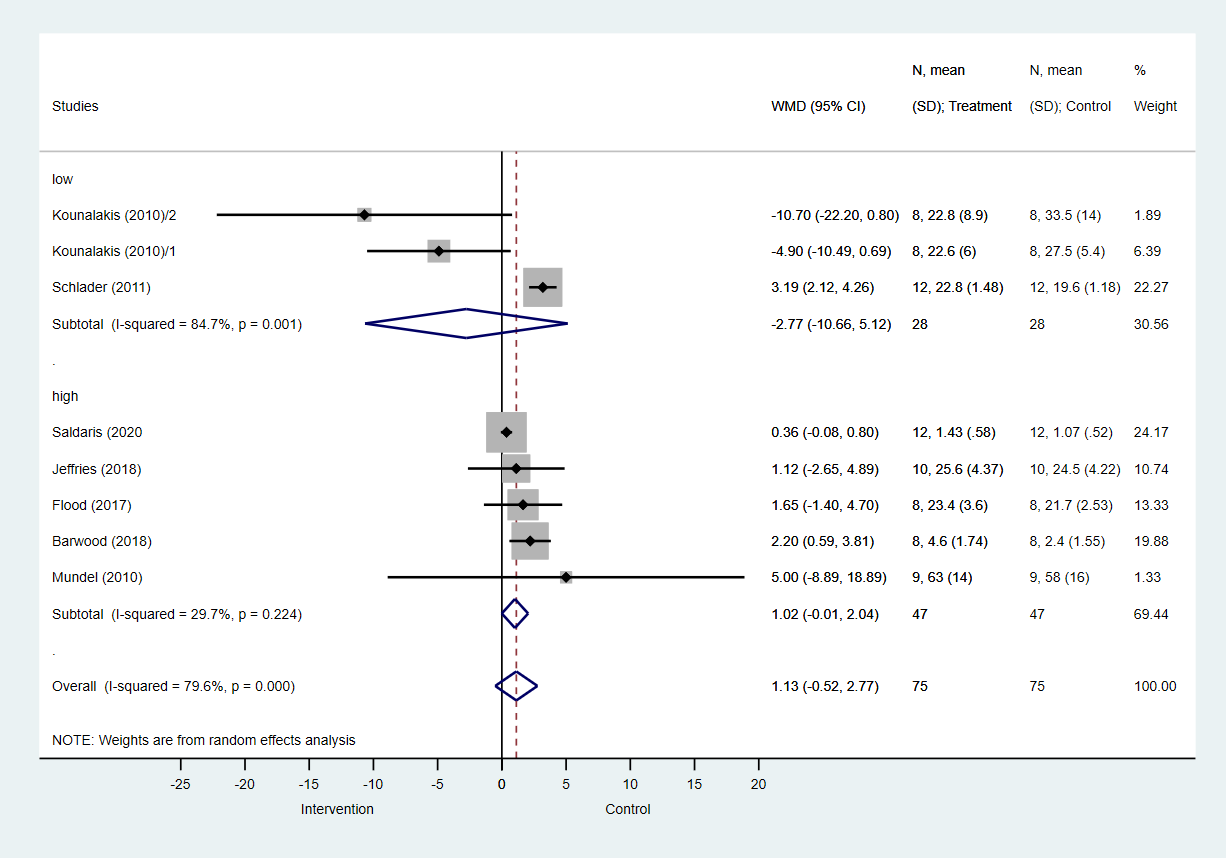


**b**

Menthol-control WMD in performance time (min)

Menthol Control

WMD (95% CI) N, mean (SD) N, mean (SD) Weight (%)

TTE
Studies

lower T_a_

higher T_a_

(2020)

(2019)

Menthol-control WMD in performance time (min)

**Figure S6.** Forest plot of the weighted mean differences (WMDs) for performance time showing the effect of menthol in (**a**) time-trial (TT) and (**b**) time-to-exhaustion (TTE) tests in subgroups of lower (< 31°C) and higher (31°C and above) ambient temperature (T_a_).

WMD

Standard error of WMD


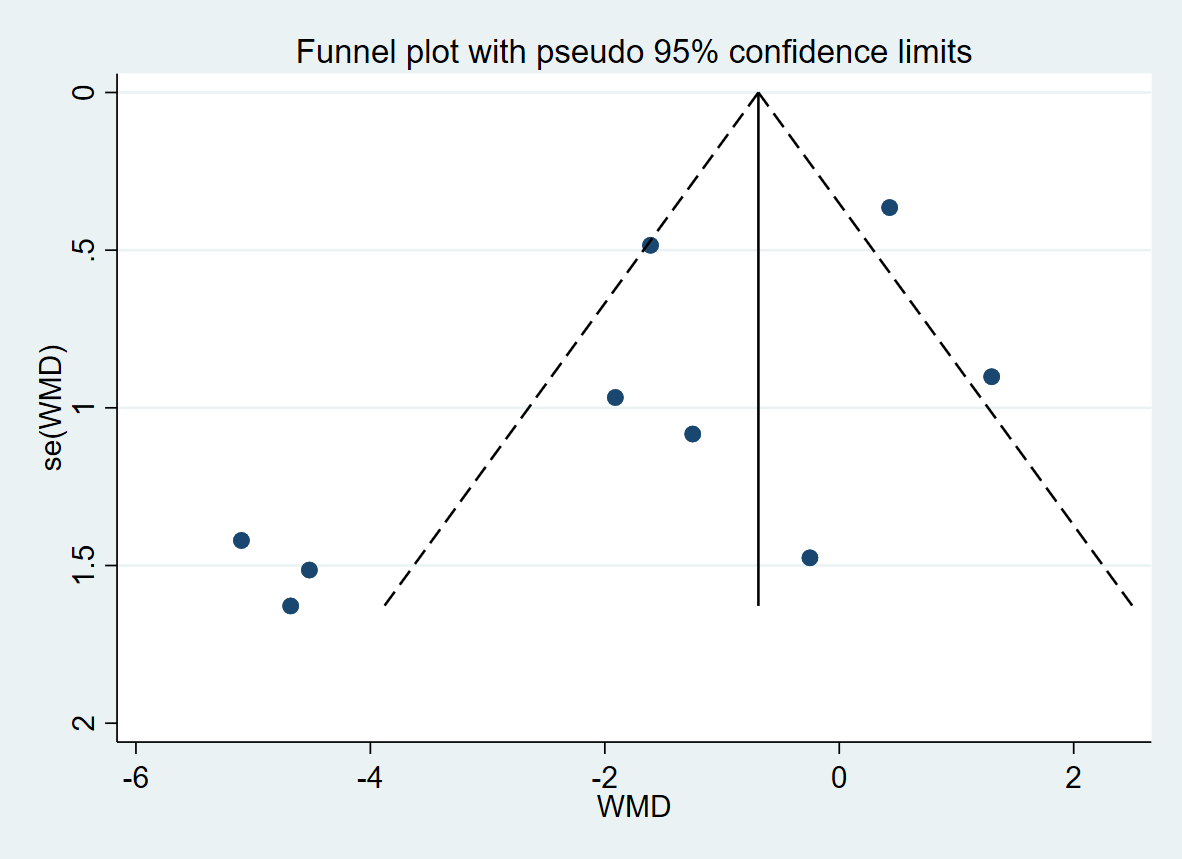


**Figure S7.** Funnel plot of the studies that were included in the forest plot of menthol’s effect on thermal sensation (n = 9, Egger’s test: p = 0.073). Here, and in Figures S8-S10, the dots represent results from studies included in the forest plot.


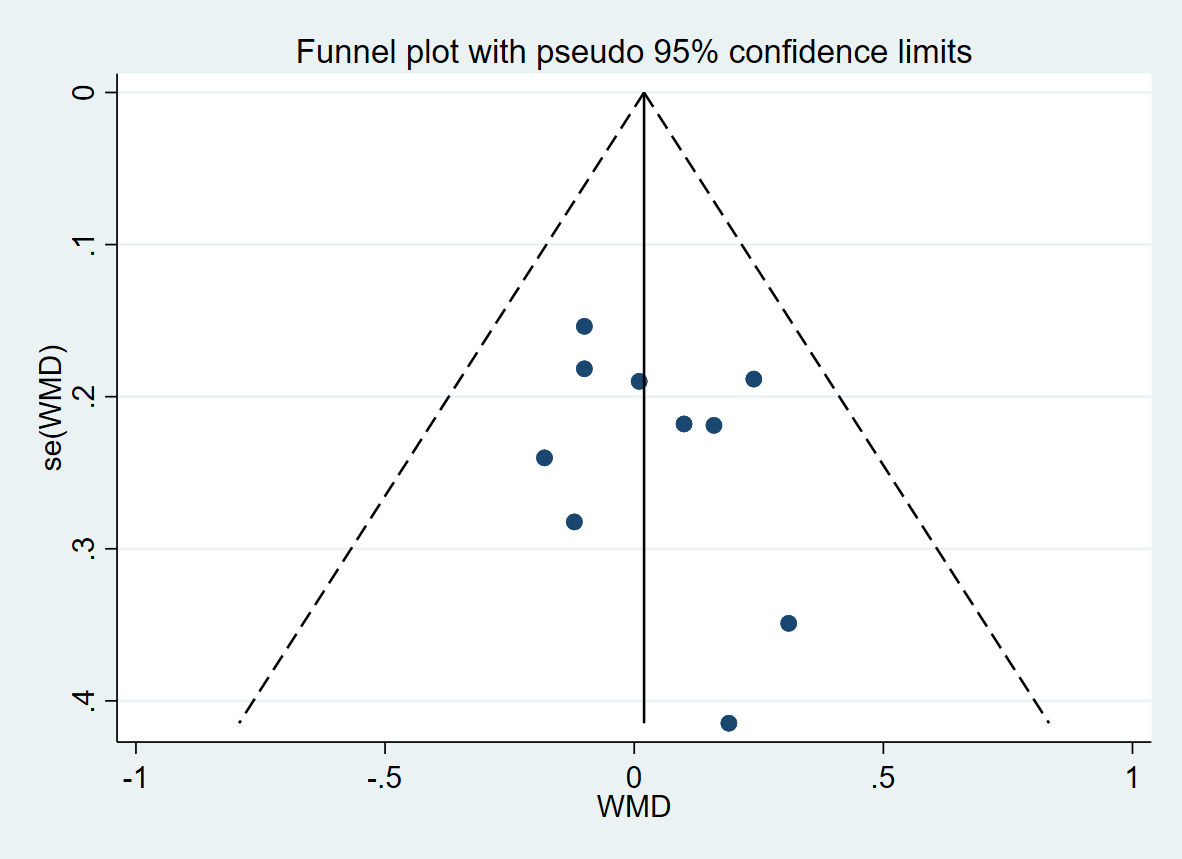


Standard error of WMD

**Figure S8.** Funnel plot of the studies that were included in the forest plot of menthol’s effect on core temperature (n = 10, Egger’s test: p = 0.341).

Standard error of WMD


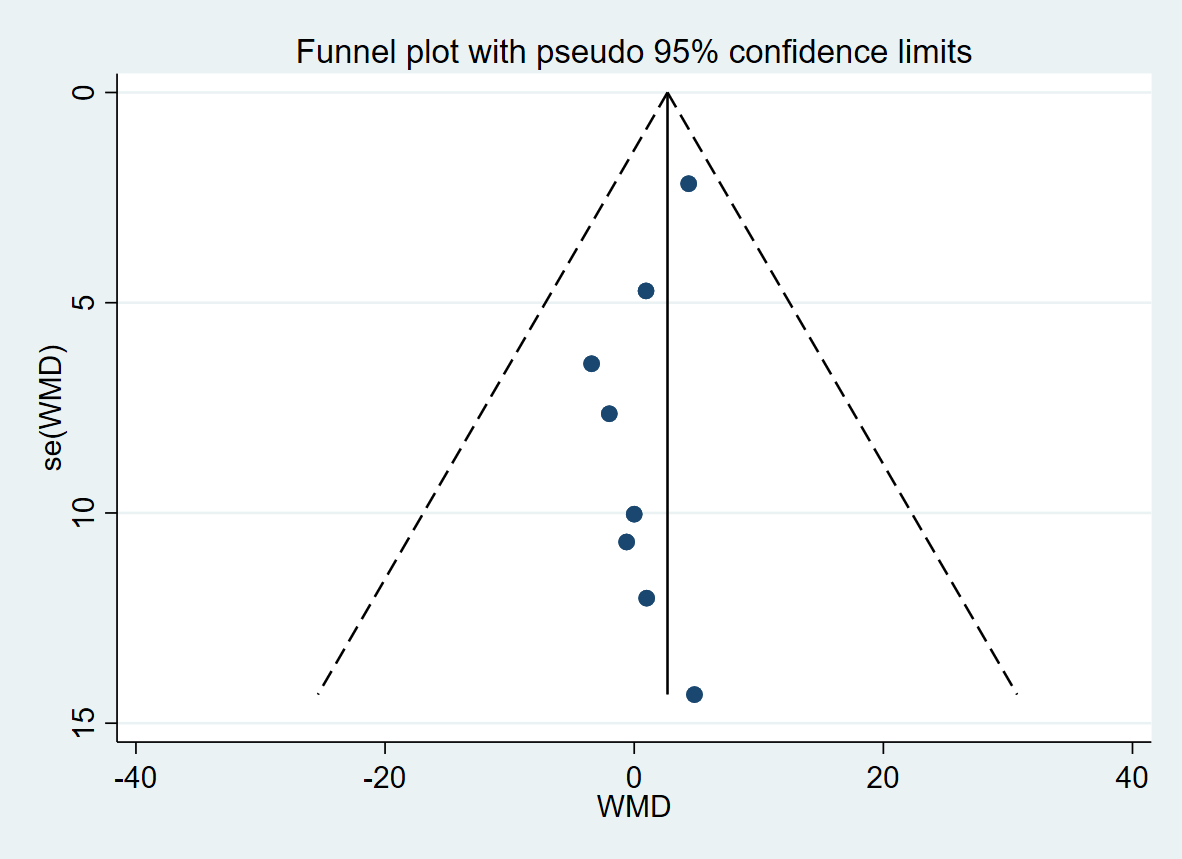


**Figure S9.** Funnel plot of the studies that were included in the forest plot of menthol’s effect on heart rate (n = 8, Egger’s test: p = 0.064).


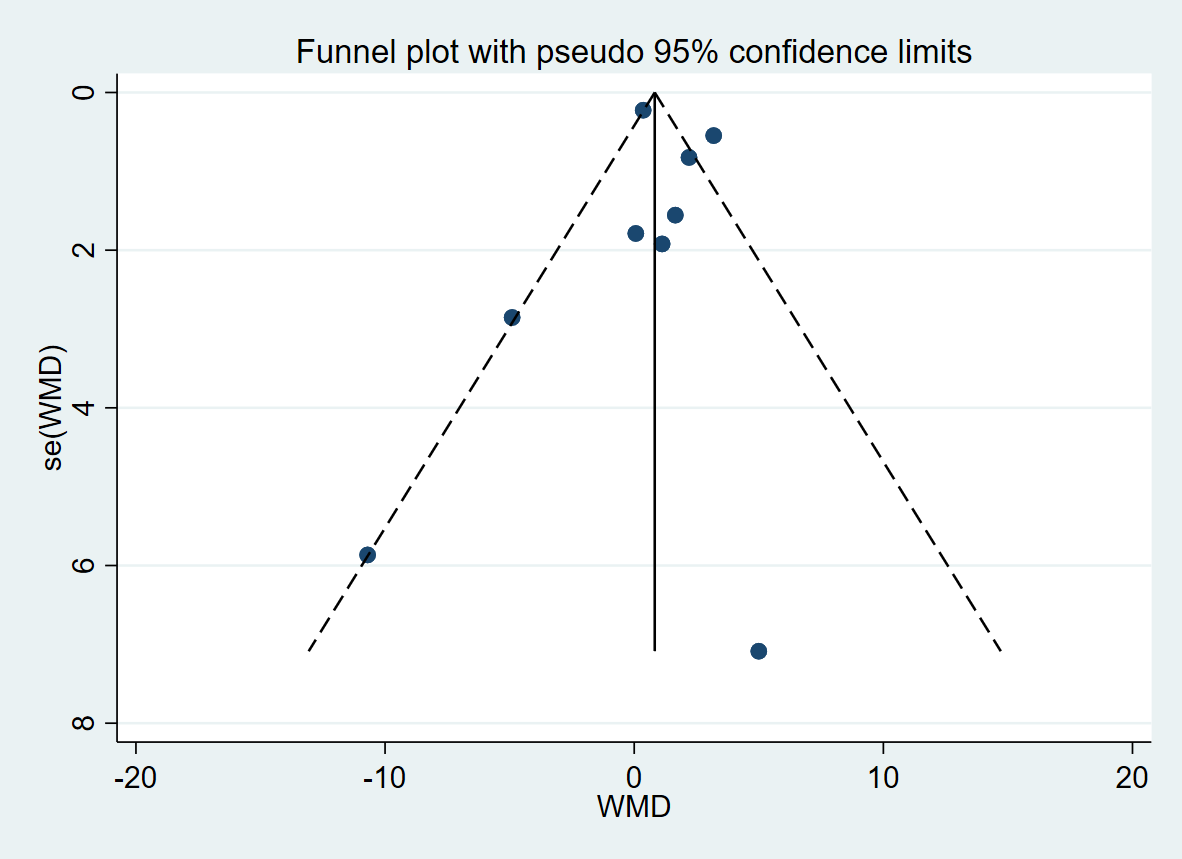


Standard error of WMD

**Figure S10.** Funnel plot of the studies that were included in the forest plot of menthol’s effect on performance time in time-to-exhaustion exercise protocols (n = 9, Egger’s test: p = 0.877).
